# Nox2 in regulatory T cells promotes angiotensin II–induced cardiovascular remodeling

**DOI:** 10.1172/JCI97490

**Published:** 2018-06-11

**Authors:** Amber Emmerson, Silvia Cellone Trevelin, Heloise Mongue-Din, Pablo D. Becker, Carla Ortiz, Lesley A. Smyth, Qi Peng, Raul Elgueta, Greta Sawyer, Aleksandar Ivetic, Robert I. Lechler, Giovanna Lombardi, Ajay M. Shah

**Affiliations:** 1 King’s College London British Heart Foundation Centre, School of Cardiovascular Medicine and Sciences, London, United Kingdom.; 2 King’s College London, Medical Research Council Centre for Transplantation, School of Immunology and Microbial Sciences, London, United Kingdom.

**Keywords:** Cardiology, Immunology, Cardiovascular disease, Hypertension, T cells

## Abstract

The superoxide-generating enzyme Nox2 contributes to hypertension and cardiovascular remodeling triggered by activation of the renin-angiotensin system. Multiple Nox2-expressing cells are implicated in angiotensin II–induced (Ang II–induced) pathophysiology, but the importance of Nox2 in leukocyte subsets is poorly understood. Here, we investigated the role of Nox2 in T cells, particularly Tregs. Mice globally deficient in Nox2 displayed increased numbers of Tregs in the heart at baseline, whereas Ang II–induced effector T cell (Teff) infiltration was inhibited. To investigate the role of Treg Nox2, we generated a mouse line with CD4-targeted Nox2 deficiency (Nox2^fl/fl^CD4Cre^+^). These animals showed inhibition of Ang II–induced hypertension and cardiac remodeling related to increased tissue-resident Tregs and reduction in infiltrating Teffs, including Th17 cells. The protection in Nox2^fl/fl^CD4Cre^+^ mice was reversed by anti-CD25 antibody depletion of Tregs. Mechanistically, Nox2^–/y^ Tregs showed higher in vitro suppression of Teff proliferation than WT Tregs, increased nuclear levels of FoxP3 and NF-κB, and enhanced transcription of CD25, CD39, and CD73. Adoptive transfer of Tregs confirmed that Nox2-deficient cells had greater inhibitory effects on Ang II–induced heart remodeling than WT cells. These results identify a previously unrecognized role of Nox2 in modulating suppression of Tregs, which acts to enhance hypertension and cardiac remodeling.

## Introduction

NADPH oxidases (Noxs) are a family of reactive oxygen species–generating (ROS-generating) enzymes with diverse pathophysiological roles (1). The prototypic member of this family, Nox2, was first identified in neutrophils, where it is involved in the killing of phagocytized microorganisms. Nox2 is also expressed in other leukocytes and many nonhematopoietic cells, such as cardiomyocytes and endothelial cells, where it modulates redox-sensitive signaling pathways (2). Cellular Nox2 activation requires the assembly of membrane-bound and cytoplasmic subunits into a multiprotein complex that transfers electrons from NADPH to molecular oxygen, a process that is stimulated by agonists such as angiotensin II (Ang II) (1).

Increased activation of the renin-angiotensin system is a major stimulus for pathological cardiovascular remodeling, contributing to hypertension, endothelial dysfunction, cardiac hypertrophy, and heart failure (3), and previous work has shown an important role for Nox2 in amplifying these processes (1, 2). Recent studies indicate that Nox2 has distinct roles in different cell types, and cell-specific contributions of endothelial cell and cardiomyocyte Nox2 to Ang II–dependent cardiovascular remodeling have been reported (4–6). It has long been known that Nox2 is also expressed in T cells (7), but relatively little is known about the T cell–specific function of Nox2 in Ang II–dependent hypertension and cardiovascular remodeling.

Previous work found that Ang II stimulates the synthesis of proinflammatory cytokines, chemokines, and adhesion molecules in the heart and vessels, inducing the recruitment and activation of T and B lymphocytes and dendritic and natural killer cells (8). In this setting, emerging evidence suggests that T cells participate in the genesis of hypertension (9). For instance, humanized mice in which the murine immune system was replaced by human leukocytes showed greater hypertension after Ang II infusion along with increased infiltration of CD4^+^ cells in the aorta and kidney (10). Circulating CD4^+^ T cells from hypertensive patients produced more IFN-γ than cells from normotensive subjects, while in mice the inhibition of the B7/CD28 costimulatory axis with CTLA-4–Ig (cytotoxic T lymphocyte–associated protein 4, also called CD152) attenuated Ang II–induced hypertension and T cell accumulation in the aorta (10, 11). Specific T cell subsets also influence cardiac remodeling; for example, CD4^+^ but not CD8^+^ T cells are reported to promote the transition from pressure overload cardiac hypertrophy to failure (12). The importance of ROS in T cell functions in hypertension has been suggested by prior studies. It was found that mice lacking T and B cells (Rag1^–/–^) displayed blunted hypertensive responses to chronic Ang II infusion, and that the hypertensive response was restored after adoptive transfer of Nox2-competent T cells (13). It was also shown that Ang II–stimulated increase in TNF-α production by T cells was inhibited by scavenging ROS (14). However, the specific role of different Nox2-containing T cell subsets remains unknown.

Here, we investigated the role of Nox2 expressed in CD4^+^ T cells in the pathophysiology of Ang II–dependent hypertension and cardiovascular remodeling. Our results identify a hitherto unrecognized role of Nox2 in CD4^+^CD25^+^FoxP3^+^ Tregs whereby Nox2 limits their suppressive activity and allows infiltration/proliferation of effector T cells (Teffs) to promote Ang II–dependent cardiovascular remodeling.

## Results

### Myocardial T cell infiltration occurs in a time-dependent manner during Ang II treatment.

**Figure 1:**
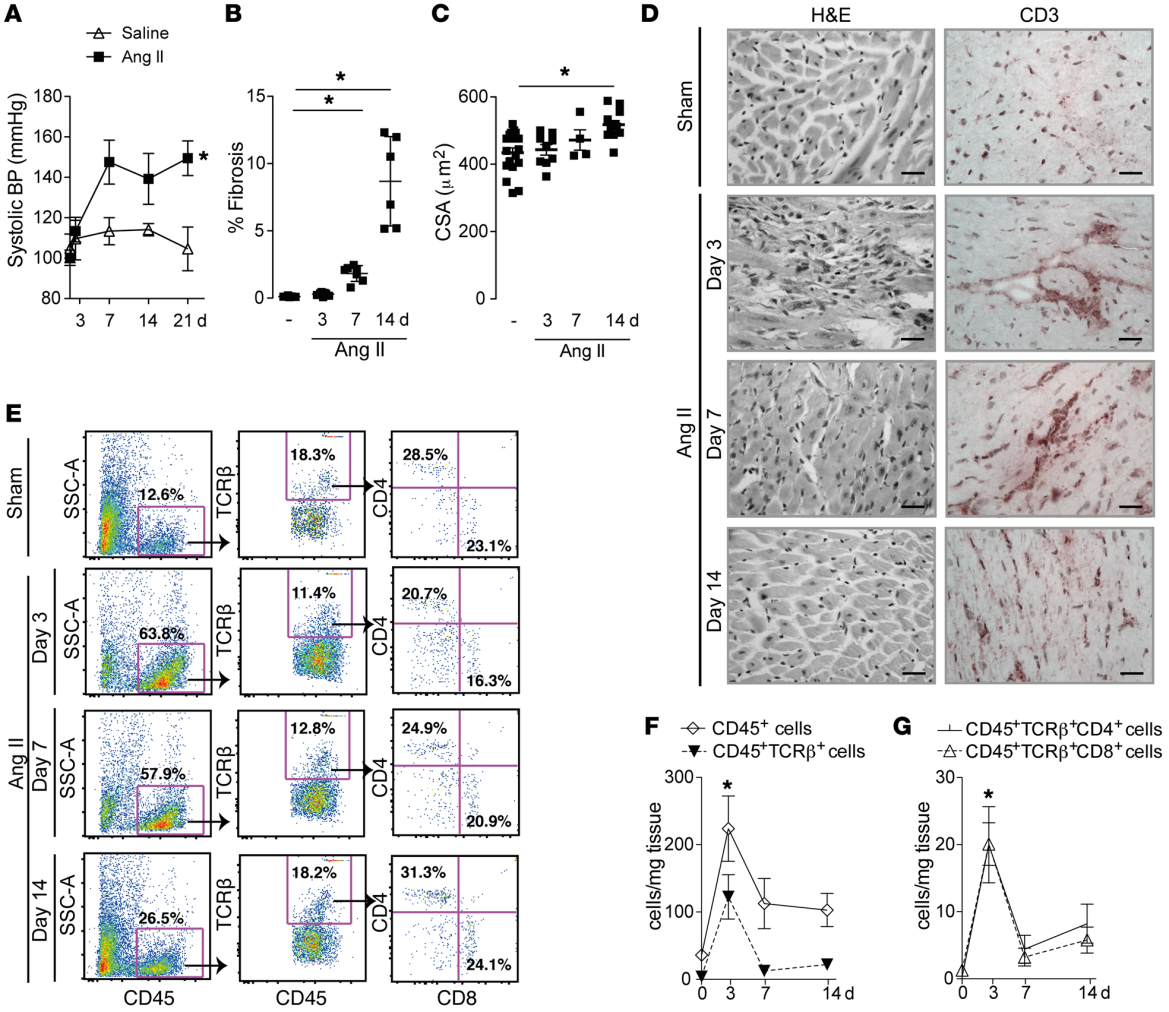
Myocardial T cell infiltration occurs during chronic Ang II infusion. WT mice were treated with Ang II infusion (1.1 mg/kg/d) or saline vehicle by osmotic minipumps. (**A**) Systolic blood pressure (BP) over 14 days of infusion. (**B**) Percentage interstitial cardiac fibrosis in myocardial sections (Picrosirius red staining). (**C**) Cardiomyocyte cross-sectional area (CSA) in myocardial sections, as an index of hypertrophy. (**D**) Myocardial sections stained with H&E (left) or immunohistochemistry using an anti-CD3 Ab (right). Scale bars: 50 μm. (**E**–**G**) Flow cytometry analyses of CD45^+^TCRβ^+^CD4^+^ and CD45^+^TCRβ^+^CD8^+^ T cells in heart digests. Representative plots are shown to the left, and mean data reported as cells per milligram tissue are shown to the right. *P < 0.05 compared with the saline group by 2-way ANOVA (**A**, **F**, and **G**) or 1-way ANOVA followed by Tukey’s post-test (**B** and **C**); *n* = 5–8 per group.

Chronic Ang II infusion (1.1 mg/kg/d for 14 days) induces significant hypertension, cardiac hypertrophy, and interstitial fibrosis in mice (Figure 1, A–C), as previously described (15, 16). A substantial component of the cardiac hypertrophy and fibrosis in this model is related to direct effects of Ang II on the heart, as these changes persist when blood pressure is normalized with the antihypertensive agent hydralazine (Supplemental Figure 1; supplemental material available online with this article; https://doi.org/10.1172/JCI97490DS1). We first studied the time course of cardiac T cell infiltration during chronic Ang II infusion. Immunostaining of cardiac sections revealed CD3^+^ cell infiltration as early as 3 days after Ang II treatment, with the cells being in perivascular clusters at days 3–7, whereas by day 14 leukocytes were mainly interstitial (Figure 1D). Flow cytometry analyses revealed a peak of CD45^+^TCRβ^+^ cells after 3 days of Ang II treatment, comprising both CD4^+^ and CD8^+^ T cells (Figure 1, E–G).

### Deficiency of Nox2 inhibits cardiac T cell infiltration in response to Ang II.

**Figure 2:**
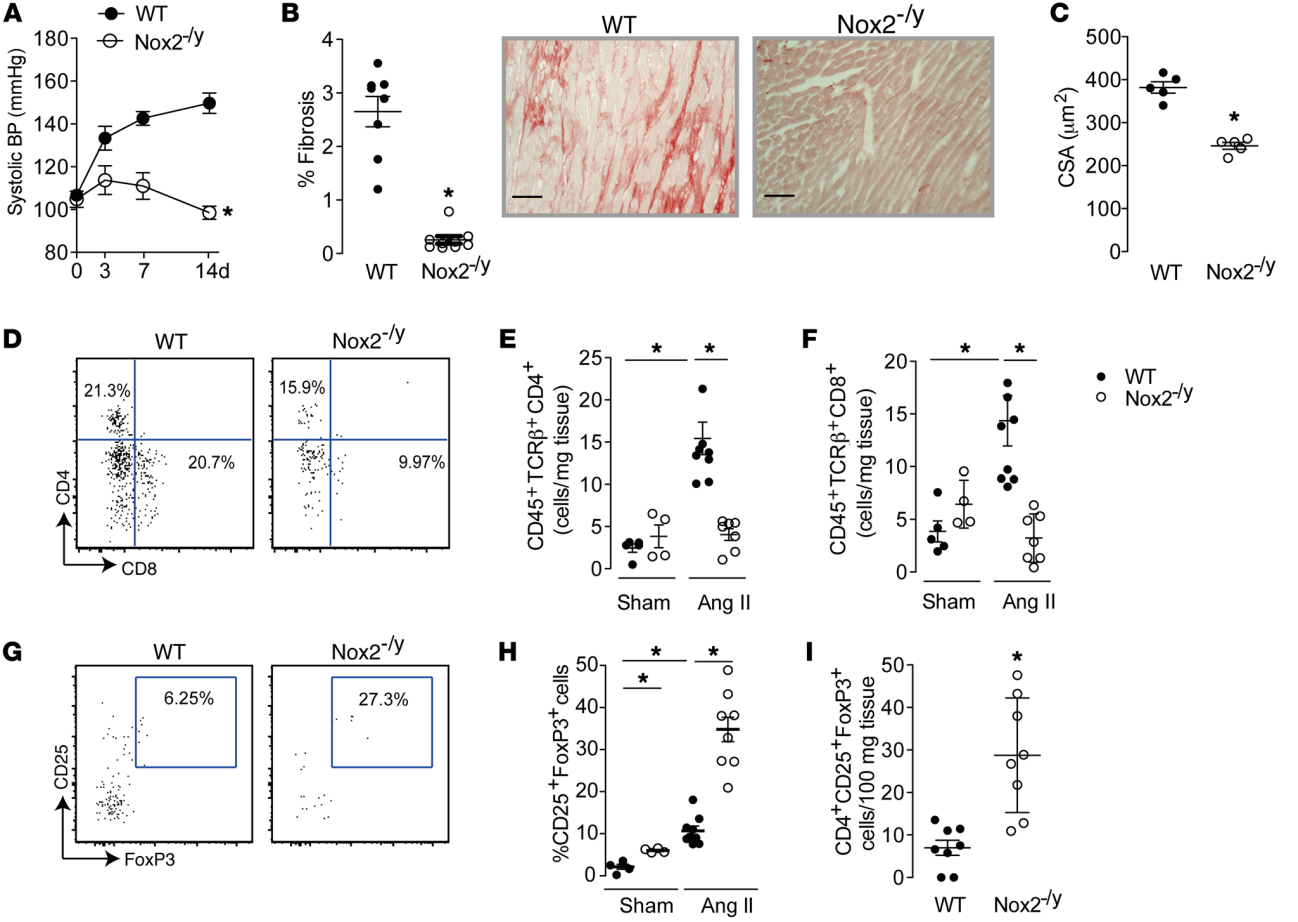
Effects of Ang II infusion on T cell infiltration in globally Nox2-deficient mice. Globally Nox2-deficient mice (Nox2^–/y^) and matched WT controls were treated with Ang II infusion (1.1 mg/kg/d). (**A**) Systolic BP was significantly lower in Nox2^–/y^ compared with WT mice. (**B**) Interstitial cardiac fibrosis after Ang II infusion. Representative myocardial sections are shown to the right. Scale bars: 50 μm. (**C**) Cardiomyocyte cross-sectional area (CSA). (**D**–**H**) Flow cytometry analyses of hearts 3 days after Ang II or saline (Sham) treatment. The numbers of CD45^+^TCRβ^+^CD4^+^ and CD45^+^TCRβ^+^CD8^+^ cells and representative plots are shown in **D**–**F**. The proportion of Tregs (CD45^+^TCRβ^+^CD4^+^CD25^+^FoxP3^+^ cells) is shown in **G**. (**H** and **I**) Flow cytometry analyses of the relative and absolute numbers of Tregs in hearts from WT and Nox2^–/y^ mice under basal conditions and after Ang II infusion. *P < 0.05 compared with the respective WT group or for the comparison shown, by 2-way ANOVA (**A**), unpaired *t* test (**B**, **C**, and **I**), or 1-way ANOVA followed by Tukey’s post-test (**E**, **F**, and **H**); *n* = 5–8 per group.

In line with previous reports (15, 16), mice globally deficient in Nox2 (Nox2^–/y^) showed attenuated hypertension, interstitial fibrosis, and cardiomyocyte hypertrophy after Ang II infusion, as compared with WT controls (Figure 2, A–C). Nox2^–/y^ mice had a substantially lower cardiac infiltration of CD4^+^ and CD8^+^ T cells after chronic Ang II infusion (Figure 2, D–F) and a higher proportion of CD4^+^CD25^+^FoxP3^+^ cells (Tregs) than WT littermates (Figure 2, G and H). Interestingly, analyses of cardiac-resident cells at baseline indicated a pronounced increase in both the proportion and the absolute numbers of Tregs in Nox2^–/y^ as compared with WT mouse hearts (Figure 2, H and I).

These results suggest that Nox2 deficiency results in enhanced Treg numbers in the heart under basal conditions and after Ang II treatment, which may limit infiltration by Teffs and cardiovascular remodeling induced by Ang II.

### In vivo role of Nox2 in CD4^+^ T cells and Tregs during Ang II infusion.

**Figure 3:**
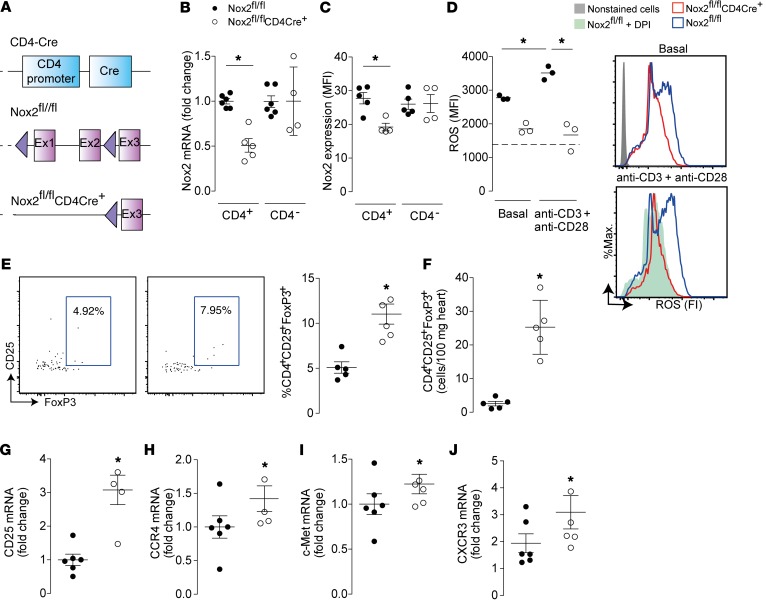
Deficiency of Nox2 in CD4^+^ T cells increases numbers of cardiac-resident Tregs. (**A**) Schematic representation of the generation of Nox2^fl/fl^CD4Cre^+^ mice. Ex, exon. (**B**) mRNA levels of Nox2 in purified CD4^+^ T cells or in total CD4^–^ cells. (**C**) Nox2 expression by flow cytometry in CD4^+^ and CD4^–^ T cells. (**D**) ROS estimated by flow cytometry of purified CD4^+^ T cells loaded with dihydroethidium after stimulation with anti-CD3 (4 μg/ml) and anti-CD28 (4 μg/ml). Representative figures are shown to the right and individual data to the left. MFI, mean fluorescence intensity. Some CD4^+^ T cells from Nox2^fl/fl^ mice were incubated with the flavoprotein Nox inhibitor diphenyleneiodonium (DPI, 1 μM) before stimulation. (**E** and **F**) Flow cytometry analyses of Tregs (CD25^+^FoxP3^+^ cells in the CD45^+^TCRβ^+^CD4^+^ population) in hearts of Nox2^fl/fl^CD4Cre^+^ and littermate Nox2^fl/fl^ mice under basal conditions. Absolute numbers of Tregs are shown in **F**. (**G**–**J**) mRNA levels of CD25, CCR4, c-Met, and CXCR3 in hearts of Nox2^fl/fl^CD4Cre^+^ and matched Nox2^fl/fl^ mice. *P < 0.05 compared with control group by 1-way ANOVA followed by Tukey’s post-test (**B**–**D**) or unpaired *t* test (**E**–**J**); *n* = 3–7 per group.

To identify the role of Nox2 in CD4^+^ T cells, we generated a novel strain of mice with a CD4-targeted Nox2 deficiency (Nox2^fl/fl^CD4Cre^+^) by crossing Nox2^fl/fl^ mice with transgenic animals expressing CD4-targeted Cre recombinase (Figure 3A). Nox2^fl/fl^CD4Cre^+^ mice appeared morphologically similar to WT littermates and were born in a normal Mendelian ratio (data not shown). Quantitative reverse transcription PCR and flow cytometry assays confirmed a significant reduction in Nox2 mRNA and protein levels in CD4^+^ T cells from Nox2^fl/fl^CD4Cre^+^ mice compared with WT littermates (Figure 3, B and C). Furthermore, stimulated CD4^+^ T cells from Nox2^fl/fl^CD4Cre^+^ mice produced less ROS than CD4^+^ T cells from WT controls, and comparable ROS levels to those observed in Nox2^fl/fl^ cells after Nox2 inhibition with the flavoprotein inhibitor diphenyleneiodonium (Figure 3D).

Under basal conditions, Nox2^fl/fl^CD4Cre^+^ mice had a significantly higher percentage and absolute number of Tregs in the heart than WT littermates (Figure 3, E and F), similar to the phenotype observed in the globally Nox2-deficient mice (Figure 2, G–I). The higher proportion of CD4^+^CD25^+^FoxP3^+^ Tregs in hearts of Nox2^fl/fl^CD4Cre^+^ mice was confirmed by augmented levels of CD25 mRNA (Figure 3G), and was accompanied by increased levels of CCR4, c-Met, and CXCR3 mRNAs (Figure 3, H–J), which may indicate an enhanced tissue tropism of Nox2-deficient as compared with WT Tregs (17). There was no difference in number of circulating CD4^+^ and CD8^+^ T cells between Nox2^fl/fl^CD4Cre^+^ mice and Nox2^fl/fl^ controls, while basal T cell numbers in the spleen were significantly higher in Nox2^fl/fl^CD4Cre^+^ mice (Supplemental Figure 2).

**Figure 4:**
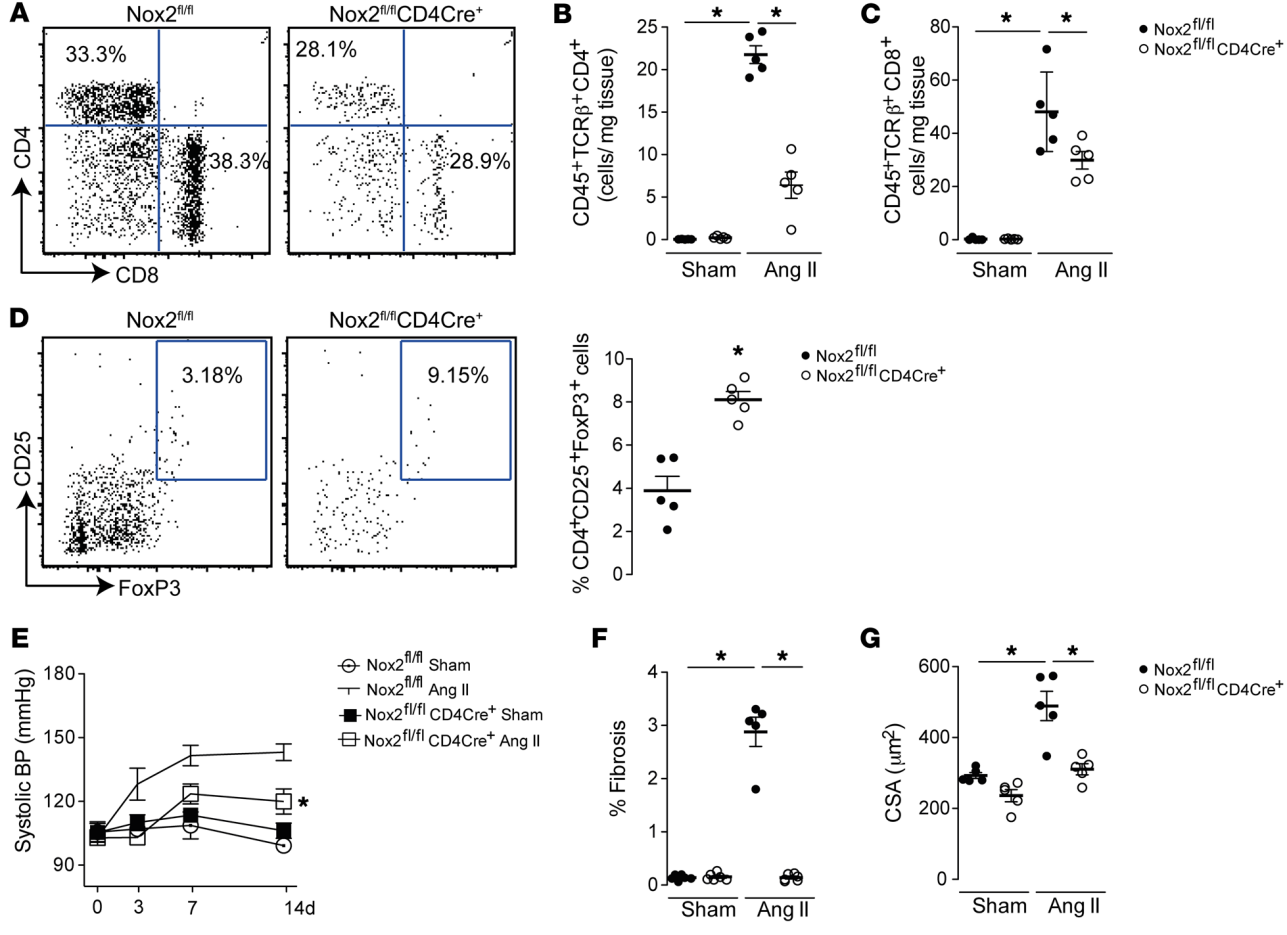
Nox2^fl/fl^CD4Cre^+^ mice are resistant to development of hypertension and heart remodeling induced by Ang II treatment. Nox2^fl/fl^CD4Cre^+^ and Nox2^fl/fl^ littermate controls were treated with Ang II (1.1 mg/kg/d) or saline (Sham) infusion. (**A**–**C**) Number of CD45^+^TCRβ^+^CD4^+^ and CD45^+^TCRβ^+^CD8^+^ T cells in heart digests by flow cytometry after 3 days of Ang II treatment. (**D**) Relative numbers of Tregs in heart digests after 3 days of Ang II treatment. Representative plots are shown to the left and mean data to the right. (**E**–**G**) Changes in systolic BP, interstitial cardiac fibrosis, and cardiomyocyte cross-sectional area (CSA) 14 days after Ang II infusion. *P < 0.05 compared with Nox2^fl/fl^ control group by 1-way ANOVA followed by Tukey’s post-test (**B**, **C**, **F**, and **G**), 2-way ANOVA (**E**), or unpaired *t* test (**D**); *n* = 5–11 per group.

After chronic Ang II infusion, Nox2^fl/fl^CD4Cre^+^ mice showed markedly lower numbers of infiltrating CD4^+^ and CD8^+^ T cells in the heart than Nox2^fl/fl^ littermates, but a higher percentage of FoxP3^+^ cells as a subset of CD4^+^ T cells (Figure 4, A–D). Similar findings were also observed in the aorta and kidneys of Nox2^fl/fl^CD4Cre^+^ mice compared with Nox2^fl/fl^ littermates (Supplemental Figure 3). The inhibition of CD4^+^ and CD8^+^ Teff infiltration was accompanied by a marked blunting of Ang II–induced hypertension, interstitial fibrosis, and cardiomyocyte hypertrophy (Figure 4, E–G). However, there was no difference in renal function between groups (Supplemental Figure 4, A–D).

**Figure 5:**
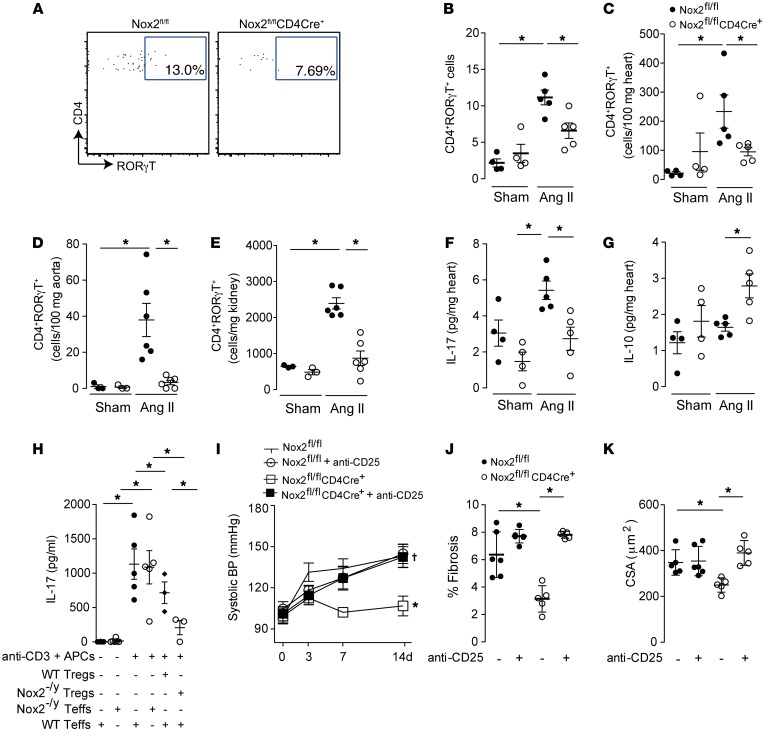
Tregs in Nox2^fl/fl^CD4Cre^+^ mice account for the inhibition of Ang II–induced hypertension and heart remodeling. Nox2^fl/fl^CD4Cre^+^ and Nox2^fl/fl^ littermate controls were treated with Ang II (1.1 mg/kg/d) or saline (Sham) infusion. (**A**–**C**) Relative and absolute numbers of CD45^+^CD4^+^RORγT^+^ (Th17) cells in heart digests by flow cytometry after 3 days of Ang II treatment. Representative plots are shown in **A**. (**D** and **E**) Absolute numbers of Th17 cells in aorta and kidney after 7 days of Ang II treatment. (**F** and **G**) Cardiac levels of IL-17 and IL-10 after 3 days of Ang II treatment. (**H**) Nox2^–/y^ Tregs inhibit IL-17 production by CD4^+^CD25^–^ cells. WT or Nox2^–/y^ CD4^+^CD25^–^ cells were stimulated with antigen-presenting cells (APCs) and anti-CD3ε Ab in the presence or absence of WT or Nox2^–/y^ Tregs for 3 days. (**I**) Systolic BP response to Ang II infusion in Nox2^fl/fl^CD4Cre^+^ and Nox2^fl/fl^ mice after treatment with anti-CD25 Ab (clone PC61, 500 μg/mouse, i.p.) to deplete Tregs. (**J** and **K**) Effect of anti-CD25 Ab treatment on interstitial cardiac fibrosis (**J**) and cardiomyocyte cross-sectional area (CSA) (**K**) in mice infused with Ang II. *P < 0.05 compared with Nox2^fl/fl^ control group, ^†^*P* < 0.05 for effect of anti-CD25 Ab in Nox2^fl/fl^CD4Cre^+^ mice, by 1-way ANOVA followed by Tukey’s post-test (**B**–**H**, **J**, and **K**) or 2-way ANOVA (**I**); *n* = 3–6 per group.

The Th17 subset of CD4^+^ T cells and IL-17 production have been implicated in the pathogenesis of Ang II–induced hypertension and remodeling, and may have reciprocal interactions with Tregs (18–20). We found that Nox2^fl/fl^CD4Cre^+^ mice had significantly lower levels of CD4^+^RORγT^+^ (Th17) cells in heart, aorta, and kidney after Ang II treatment (Figure 5, A–E). Additionally, the levels of IL-17 were lower and those of IL-10 higher in Nox2^fl/fl^CD4Cre^+^ mouse hearts compared with Nox2^fl/fl^ controls after Ang II infusion (Figure 5, F and G), consistent with a switch in Th17/Treg balance from pro- to antiinflammatory. To assess whether Nox2 deficiency in Teffs directly impacts on IL-17 production or whether Nox2-deficient Tregs indirectly inhibit IL-17–producing cells, we undertook coculture studies. CD4^+^CD25^–^ cells (Teffs) from WT or Nox2^–/y^ mice cultured with antigen-presenting cells (APCs) in the presence of anti-CD3ε antibody (Ab) produced similar levels of IL-17 (Figure 5H). However, WT Teffs cocultured with Nox2^–/y^ Tregs produced significantly lower levels of IL-17 than WT Teffs cocultured with WT Tregs (Figure 5H), suggesting that Nox2-deficient Tregs more efficiently block IL-17 production than WT Tregs. To define the specific role of Nox2-deficient Tregs in vivo, we depleted Tregs in Nox2^fl/fl^CD4Cre^+^ mice by treatment with PC61 monoclonal anti-CD25 antibody 1 day before commencing Ang II infusion (Supplemental Figure 5). The protection against Ang II–induced remodeling was abolished in anti-CD25–treated Nox2^fl/fl^CD4Cre^+^ mice, and the animals developed a similar level of hypertension, hypertrophy, and fibrosis to that observed in WT mice undergoing Ang II infusion (Figure 5, I–K). Treatment with anti-CD25 Ab in control Nox2^fl/fl^ mice did not significantly alter the level of Ang II–induced hypertension.

In addition to CD4^+^CD25^+^FoxP3^+^ Tregs (also known as conventional Tregs), CD8^+^FoxP3^+^ Tregs may also contribute to antiinflammatory effects. We found that the proportion of CD8^+^FoxP3^+^ Tregs was significantly higher in hearts and aorta of Ang II–treated Nox2^fl/fl^CD4Cre^+^ mice than control Nox2^fl/fl^ mice (Supplemental Figure 6, A and B). Since CD4 is expressed in double-positive T cells during development in the thymus, this observation may reflect targeting of Nox2 in CD8^+^ T cells in Nox2^fl/fl^CD4Cre^+^ mice. Indeed, Nox2 expression was reduced in CD8^+^ and CD4^+^CD8^+^ T cells in the thymus of Nox2^fl/fl^CD4Cre^+^ mice (Supplemental Figure 6C).

Taken together, these results indicate that Nox2 deficiency in Tregs limits Ang II–induced hypertension and cardiac remodeling.

**Figure 6:**
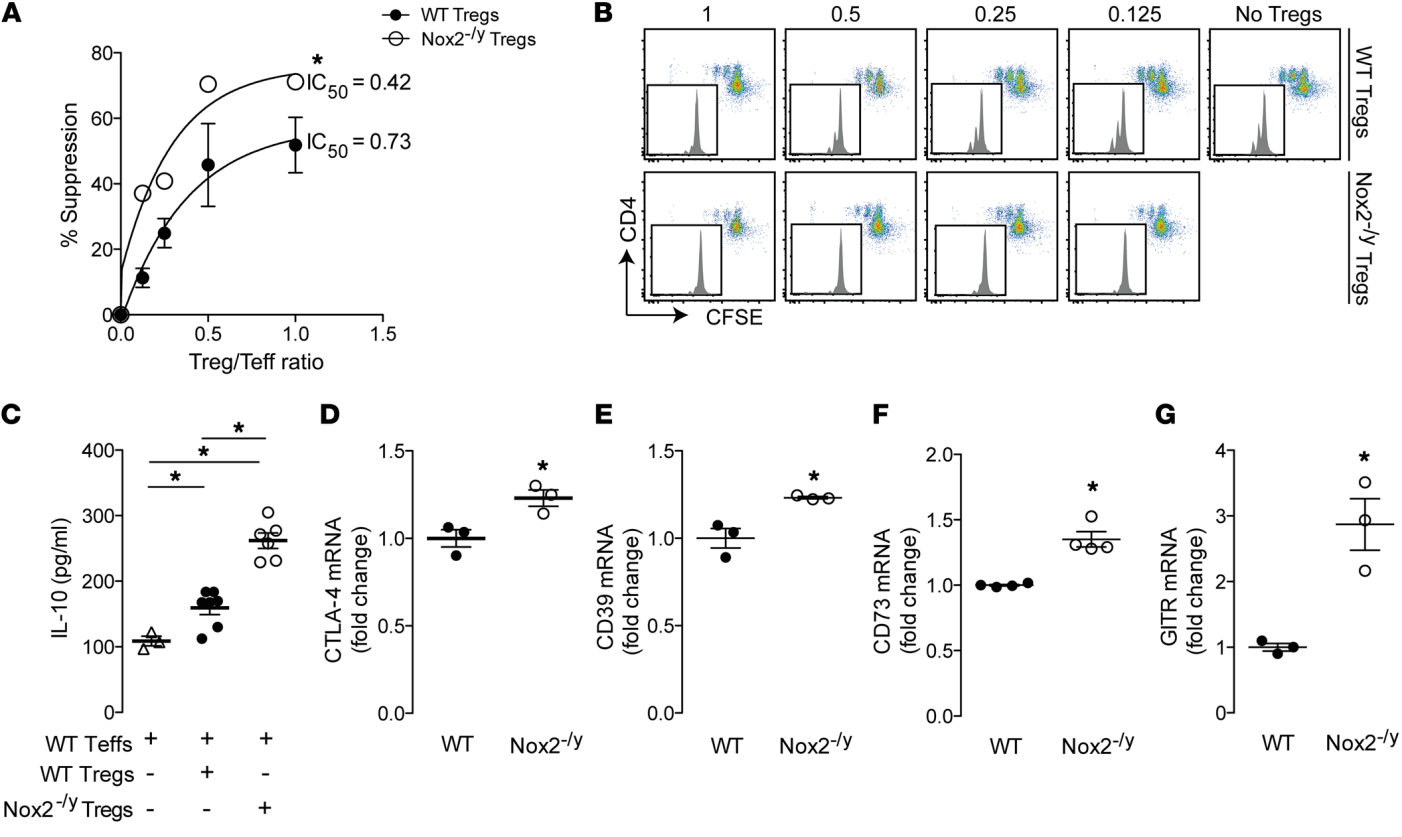
Tregs deficient in Nox2 are more suppressive than WT Tregs. (**A** and **B**) In vitro suppression assay using Tregs purified from spleen and lymph nodes of Nox2-deficient mice (Nox2^–/y^) and WT littermate controls. Cells were stimulated with APCs and anti-CD3ε Ab. Representative plots in **B** show proliferation of Teffs after 3 days of stimulation; numbers at the top are the ratio of Tregs to Teffs. Mean data are shown in **A**. The “IC_50_” is the ratio of Tregs/Teffs at which there was 50% suppression of Teff proliferation. (**C**) Levels of IL-10 in culture supernatants determined by cytometric bead array. The cell combinations that were cocultured are shown at the bottom. (**D**–**G**) Baseline levels of mRNA for CTLA-4, CD39, CD73, and GITR in Tregs purified from spleen and lymph nodes. *P < 0.05 for highlighted comparisons, by 2-way ANOVA (**A**), 1-way ANOVA followed by Tukey’s post-test (**C**), or unpaired *t* test (**D**–**G**); *n* = 3–6 per group.

*Nox2-deficient Tregs are more suppressive than WT Tregs and have increased nuclear levels of FoxP3 and NF-*κ*B activation*. To investigate mechanisms underlying the protective effect of Nox2 deficiency in Tregs against Ang II–induced pathology, we first studied the impact of Nox2 deficiency on the function of CD4^+^CD25^+^ Tregs. In vitro suppression assays revealed that Nox2-deficient CD4^+^CD25^+^ Tregs were more efficient at inhibiting Teff proliferation induced by anti-CD3ε plus APCs as compared with WT CD4^+^CD25^+^ Tregs (Figure 6, A and B). Moreover, cocultures of Nox2-deficient CD4^+^CD25^+^ Tregs and WT CD4^+^ Teffs had higher levels of the antiinflammatory cytokine IL-10 in supernatants than cocultures of WT Tregs and WT Teffs (Figure 6C). There was no difference in proliferation between Nox2-deficient and WT Teffs (Supplemental Figure 7A). In cocultures of WT Teffs/WT Tregs compared with WT Teffs/Nox2-deficient Tregs, there was no difference in the levels of TNF-α and IFN-γ in supernatants (Supplemental Figure 7, B and C), showing that Nox2 deficiency in Tregs does not affect TNF-α and IFN-γ production by Teffs although it reduces IL-17 production (Figure 5H).

Tregs suppress through different mechanisms, including the release of antiinflammatory cytokines (IL-10 and TGF-β) (21), inhibition of dendritic cell costimulation (CTLA-4–mediated antagonism of CD28 or CTLA-4 removing CD80/CD86 from APCs by transendocytosis) (22), synthesis of adenosine by coordinated activity of the ectoenzymes CD39 and CD73 (23, 24), and direct induction of apoptosis of CD4^+^ or CD8^+^ Teffs (25). We found that Nox2-deficient Tregs expressed higher levels of mRNA for CTLA-4, CD39, and CD73 than WT Tregs (Figure 5, D–F). Additionally, Nox2-deficient Tregs expressed higher mRNA levels of the glucocorticoid-induced TNF-related receptor (GITR) (Figure 5G), which has previously been shown to be related to proliferation and maintenance of the suppressive phenotype of Tregs (26). Increased protein levels of CTLA-4, CD39, CD73, and GITR in Nox2-deficient versus WT Tregs were further confirmed by flow cytometry (Supplemental Figure 8).

**Figure 7:**
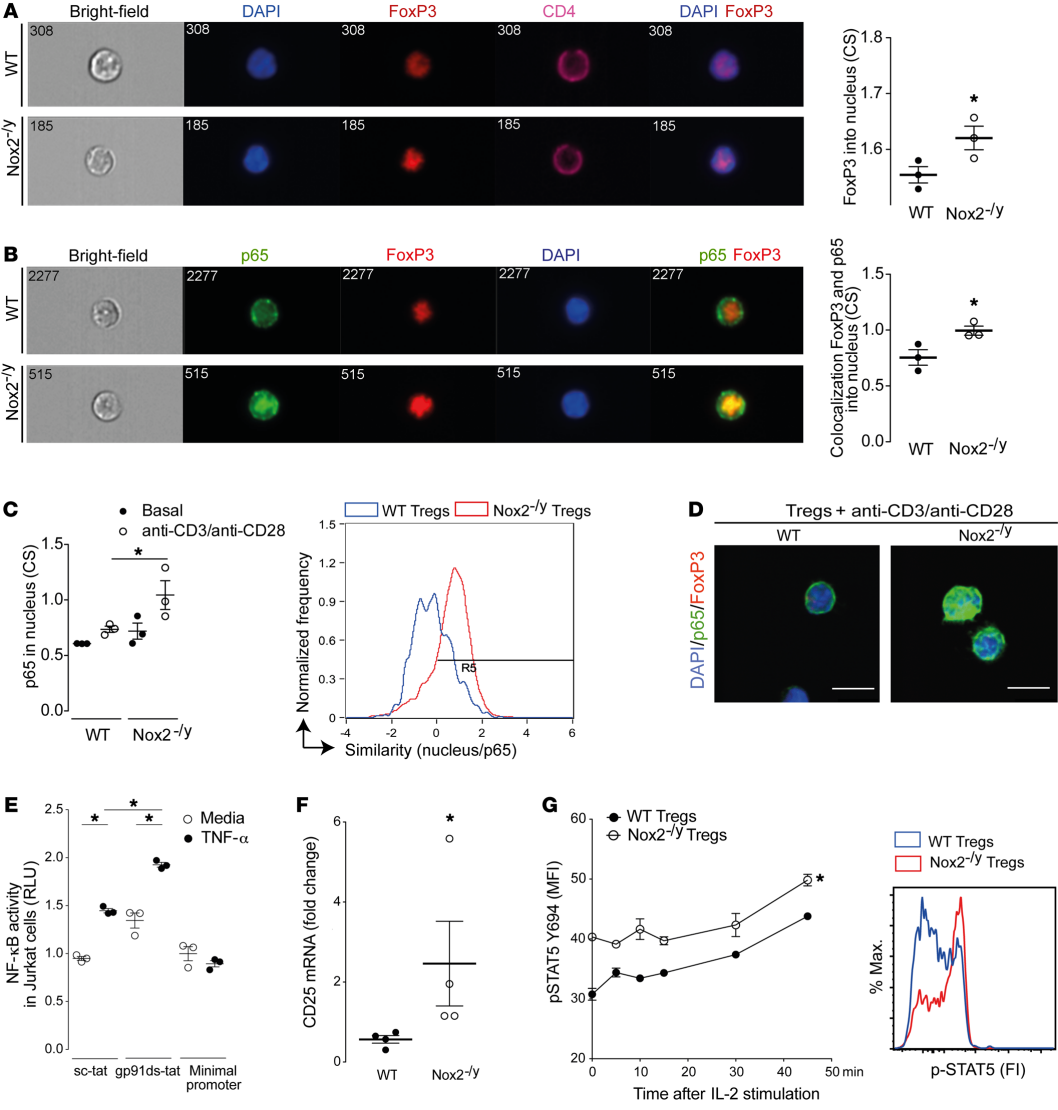
Deficiency of Nox2 in Tregs increases nuclear levels of FoxP3 and NF-κB activation. (**A**) Representative ImageStream pictures (left) and mean similarity scores (right) for the colocalization of FoxP3 and DAPI in CD4^+^FoxP3^+^ cells (Tregs) under basal conditions. (**B**) Representative ImageStream pictures (left) and mean similarity scores (right) for the colocalization of FoxP3 and p65 in Tregs after anti-CD3 plus anti-CD28 stimulation. (**C**) Nuclear localization of p65 in Tregs after anti-CD3 plus anti-CD28 stimulation. CS, coefficient of similarity. Representative overlay histogram of similarity of p65/DAPI in WT Tregs and Nox2^–/y^ Tregs is shown to the right. (**D**) Colocalization of FoxP3 and p65 in the nucleus of Tregs by confocal microscopy. Scale bars: 7.5 μm. (**E**) NF-κB transcriptional activity assessed by a luciferase promoter assay in Jurkat cells preincubated with a specific Nox2 peptide inhibitor, gp91ds-tat (gp91ds, 30 μM), or a scrambled peptide control, scrambled-tat (sc-tat; 30 μM). Cells transfected with minimal promoter and thymidine kinase Renilla were used as controls. RLU, relative lumen units. (**F**) mRNA levels of CD25 in purified Tregs (CD4^+^CD25^+^). (**G**) Level of STAT5 phosphorylation (p-STAT5, Y694) assessed in purified Tregs (CD4^+^CD25^+^FoxP3^+^) by flow cytometry after 30 minutes of IL-2 (100 IU/ml) stimulation. Representative histogram shown to the right. MFI, mean fluorescence intensity. *P < 0.05 for highlighted comparisons by unpaired *t* test (**A**, **B**, and **F**), 2-way ANOVA (**G**), or 1-way ANOVA followed by Tukey’s post-test (**C** and **E**); *n* = 3 independent experiments except where shown otherwise.

Substantial evidence indicates that the forkhead box P3 transcription factor, FoxP3, plays a critical role in the development and function of CD4^+^CD25^+^ Tregs. Mutations of human FoxP3 result in dysfunction or impaired development of Tregs and lead to immunodysregulation and diverse immune disorders (27, 28). Here, we first observed by imaging flow cytometry, confocal microscopy, and immunoblotting that Tregs deficient in Nox2 had increased nuclear levels of FoxP3 under basal conditions (Figure 7A and Supplemental Figure 9). It was previously reported that FoxP3 associates with the NF-κB subunit p65 (also known as RelA) on the CD25 promoter, mediating the transcription of the CD25 gene (also known as IL-2 receptor α chain or IL2RA) (29), and that an NF-κB–dependent transcriptional program promotes Treg identity and function (30). In addition, we recently demonstrated that the deficiency of Nox2 increases nuclear NF-κB activation in myeloid cells, secondary to changes in nuclear redox state (31). In line with these reports, we observed an enhanced colocalization of FoxP3 and p65 in stimulated Nox2-deficient versus WT Tregs (Figure 7B). Moreover, Nox2-deficient Tregs had higher nuclear p65 levels after anti-CD3 plus anti-CD28 stimulation than WT Tregs, as evaluated by imaging flow cytometry and confocal microscopy (Figure 7, C and D). More direct assessment of NF-κB transcriptional activity was undertaken in Jurkat cells transfected with an NF-κB firefly reporter construct. In these cells, the inhibition of Nox2 by preincubation with gp91ds-tat (a selective peptide inhibitor of Nox2) resulted in a higher NF-κB activation after TNF-α stimulation than in cells preincubated with a scrambled peptide control (scrambled-tat) (Figure 7E). Finally, we found that Nox2-deficient Tregs expressed higher levels of CD25 mRNA (Figure 7F), consistent with the increased nuclear levels of FoxP3 and p65. Also, there was enhanced phosphorylation of STAT5 after stimulation with IL-2 (as a readout of functional CD25 activity) in Nox2-deficient Tregs compared with WT Tregs (Figure 7G).

Therefore, the increased suppressive activity of Nox2-deficient Tregs may be related to their increased nuclear levels of FoxP3 and enhanced NF-κB activation, which drives an increased expression of CD25 and other molecules mediating suppression of Teffs.

### Adoptive transfer of Nox2-deficient Tregs inhibits Ang II–induced hypertension and heart remodeling.

**Figure 8:**
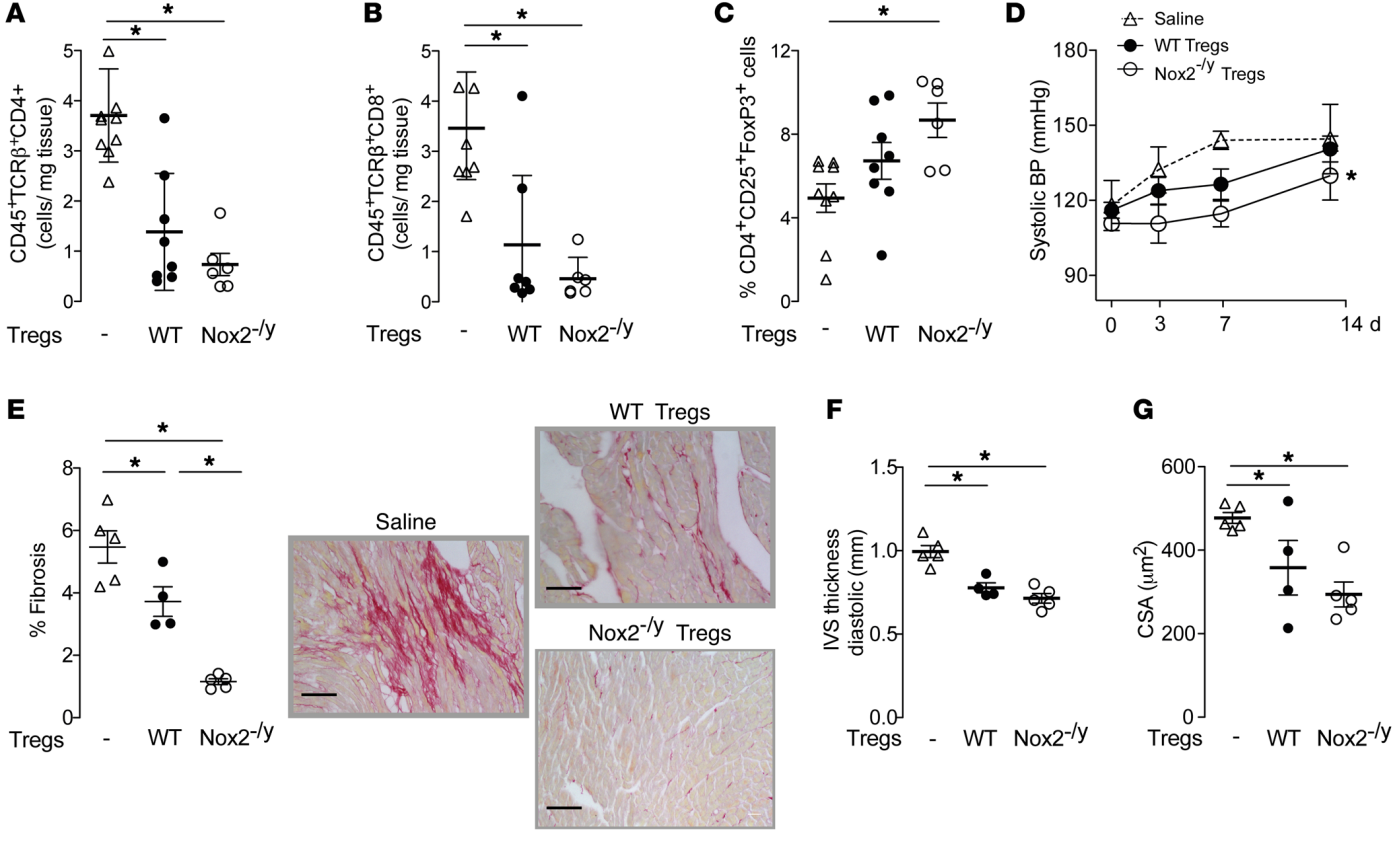
Effect of adoptive transfer of Nox2-deficient or WT Tregs on the response to Ang II infusion. WT mice were treated with Ang II infusion (1.1 mg/kg/d, 14 days) by osmotic minipump. Immediately before minipump implantation, mice received 1 × 10^6^ WT or Nox2^–/y^ Tregs or saline control by i.v. injection. (**A**–**C**) Absolute numbers of CD45^+^TCR^+^CD4^+^ and CD45^+^TCR^+^CD8^+^ T cells and relative numbers of Tregs (CD25^+^FoxP3^+^) in the heart after 14 days of Ang II infusion. (**D**) Effect of adoptive transfer of Tregs on systolic BP. (**E**) Interstitial cardiac fibrosis in myocardial sections. Representative photomicrographs are shown to the right (scale bars: 50 μm) and mean data to the left. (**F**) Echocardiographic interventricular septal thickness (IVS) as a marker of left ventricular hypertrophy. (**G**) Cardiomyocyte cross-sectional area (CSA) in myocardial sections. *P < 0.05 for highlighted comparisons by 1-way ANOVA followed by Tukey’s post-test (**A**–**C** and **E**–**G**) or 2-way ANOVA (**D**); *n* = 5–8 per group.

To validate the importance of Nox2 in Tregs in Ang II–induced cardiovascular remodeling, we performed adoptive transfer studies in which WT Tregs or Nox2^–/y^ Tregs were injected into WT mice immediately before initiation of Ang II infusion. Animals treated with either WT or Nox2^–/y^ Tregs had lower numbers of CD4^+^ and CD8^+^ T cells in the heart as compared with saline-treated controls (Figure 8, A and B). Mice that received Nox2^–/y^ Tregs had a higher percentage of Tregs in the heart after Ang II treatment than mice injected with WT Tregs (Figure 8C). Adoptive transfer of Tregs, either WT or Nox2^–/y^, inhibited Ang II–induced hypertension, cardiac fibrosis, and cardiomyocyte hypertrophy (Figure 8, D–G). Notably, the adoptive transfer of Nox2-deficient Tregs induced a greater inhibition of Ang II–induced hypertension and heart fibrosis than was observed with the adoptive transfer of WT Tregs (Figure 8, D and E). Therefore, Tregs deficient in Nox2 are more protective than WT Tregs in the setting of hypertension and cardiac remodeling.

## Discussion

**Figure 9:**
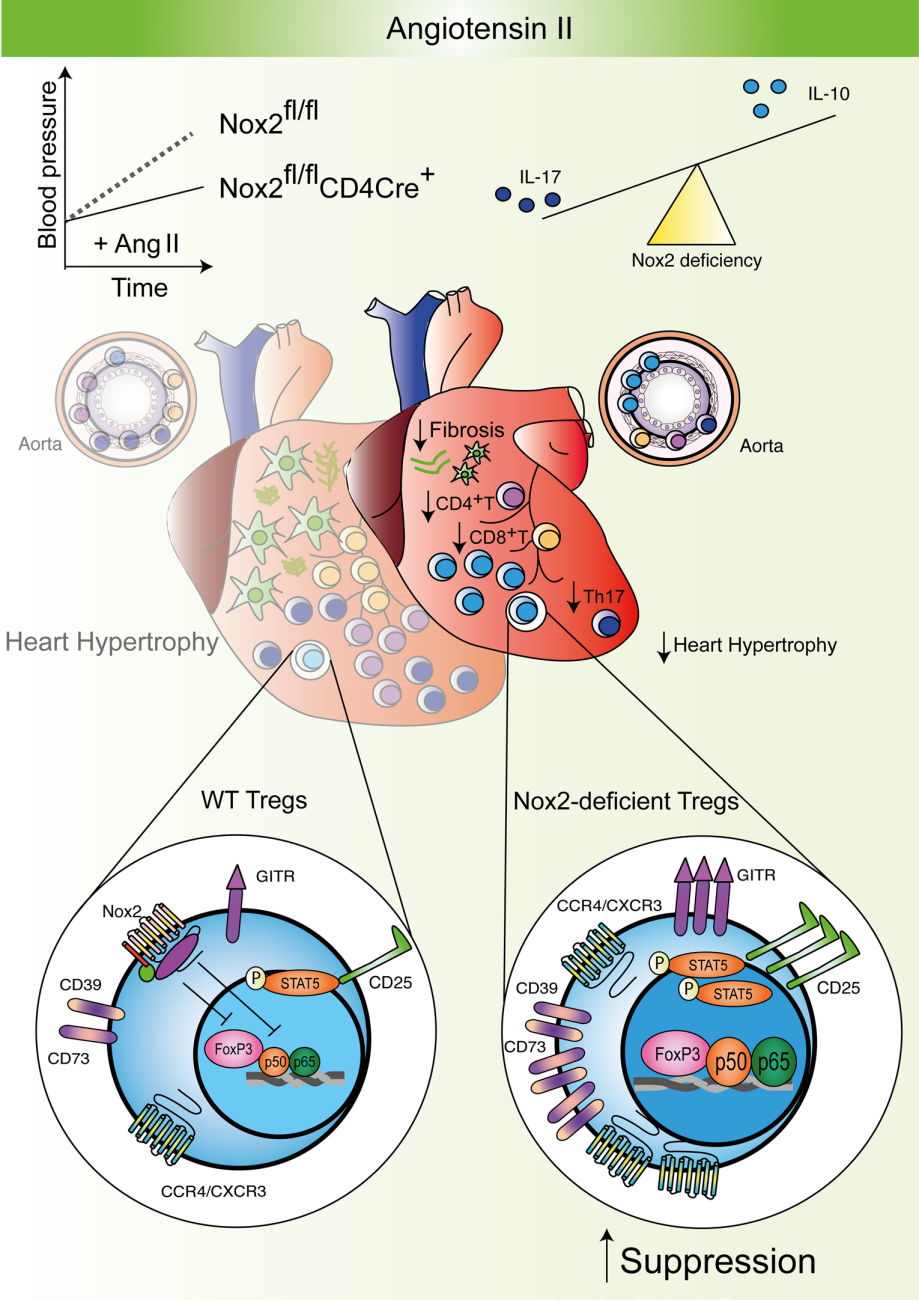
Schematic of cellular and molecular mechanisms through which Nox2 in CD4^+^ T cells regulates Ang II–induced hypertension and heart remodeling. Deficiency of Nox2 in Tregs results in increased numbers of Tregs in the heart and vessels and protects against the development of hypertension, interstitial cardiac fibrosis, and cardiomyocyte hypertrophy. Mechanistically, the deficiency of Nox2 in Tregs enhances their suppressive function through an increase in nuclear levels of FoxP3 and NF-κB activation, and higher levels of GITR, CTLA-4, CD39, CD73, and CD25. The higher CD25 levels may drive an increased STAT5 phosphorylation under IL-2 stimulation, and lead to a positive feedback promoting suppression. The deficiency of Nox2 also drives a change in balance between Tregs and Th17 cells to a more antiinflammatory profile, with higher IL-10 and lower IL-17 levels.

The major novel finding of this study is that the expression of Nox2 in CD4^+^CD25^+^FoxP3^+^ Tregs plays a vital role in their function to orchestrate Ang II–induced hypertension and cardiac remodeling. We show that Nox2 in Tregs limits their suppressive activity and therefore allows an increase in the infiltration/proliferation of Teffs, including Th17 cells, which enhances Ang II–induced hypertension, cardiac fibrosis, and hypertrophy. Mechanistically, the higher suppressive activity of Nox2-deficient Tregs involves enhanced nuclear levels of FoxP3 and NF-κB activation, which may drive the expression of the IL-2 receptor CD25 and its downstream signaling via STAT5 phosphorylation (Figure 9). Despite Nox2 being expressed in multiple cell types that are potentially involved in Ang II–dependent pathology, we find that targeted inhibition of Nox2 in Tregs is sufficient to significantly ameliorate Ang II–induced hypertension and heart remodeling.

T cells express the Ang II receptor AT1, which is known to enhance T cell proliferation and modulate inflammatory responses (32, 33). The importance of T cells in the pathogenesis of hypertension has been recognized for many years, with most studies focusing on the role of T cells in aggravating hypertension, vascular remodeling, and renal dysfunction (10, 11, 13, 34). Moreover, the contribution of T cells to cardiac remodeling has been increasingly recognized during hypertension or pressure overload (12, 35). More recently, it has been found that Tregs (CD4^+^CD25^+^FoxP3^+^) can act to limit Ang II–induced inflammation and damage in the vasculature and heart, thereby reducing the extent of hypertension and cardiac remodeling (36–40). However, the mechanisms that control these suppressive actions of Tregs in hypertension and cardiac remodeling were unclear. In the present study, we observe that when Nox2 is deficient in Tregs, they are more suppressive in vitro, accumulate in the heart at baseline, and are protective against Ang II–induced hypertension and heart remodeling.

We investigated the mechanisms that may underlie the greater suppressive effects of Nox2-deficient compared with WT Tregs (defined as CD4^+^CD25^+^FoxP3^+^ cells). Nox2-deficient Tregs are found to have increased expression levels of CTLA-4, CD39, CD73, and GITR, which are all involved in suppression through different mechanisms (22–24). Nox2-deficient Tregs also exhibit higher nuclear levels of FoxP3 and the p65 subunit of NF-κB, which could drive the transcription of CD25. Indeed, previous studies have shown that FoxP3, induced by CD28 signaling in human CD4^+^ T lymphocytes, synergizes with p65 on a regulatory region of the CD25 promoter to mediate the transcriptional activation of the CD25 gene (29). We find increased CD25 expression in Nox2-deficient Tregs, and, in keeping with this, these cells have higher levels of phosphorylated STAT5 compared with WT Tregs after IL-2 stimulation. The crucial role of IL2R/STAT5 signaling in Treg-suppressive function has recently been highlighted by the demonstration that ablation of STAT5 signaling compromised Treg suppression in vitro and in vivo (41). Moreover, Tregs with enhanced STAT5 activation express higher levels of proteins involved in cell adhesion, which results in higher interaction with dendritic cells and blockade of costimulatory molecule synthesis. It is also well established that STAT5 activation in Tregs correlates with FoxP3 expression and their proliferative capacity (41), which in Nox2-deficient Tregs might generate a positive feedback of suppression.

The enhanced NF-κB activation in Nox2-deficient Tregs is rather analogous to findings that we and others have reported in macrophages, where Nox2 deficiency results in hyperactivation of NF-κB in response to lipopolysaccharide (31, 42). We showed that the increased NF-κB activation is related to a Nox2-dependent regulation of nuclear redox state (31), and it is conceivable that a similar mechanism may contribute to the increase in nuclear p65 and FoxP3 levels observed in the current study. Previous work has shown the importance of NF-κB in the development and suppressive function of Tregs, at least in part through the regulation of transcription of FoxP3 and CD25 (29, 30, 43, 44). Therefore, it is likely that the increased nuclear levels of FoxP3 and NF-κB in Nox2-deficient Tregs contribute to their higher suppressive capacity.

Interestingly, at least part of the effect of Nox2-deficient Tregs is related to a greater inhibition of Th17 cells, which are found in reduced numbers in Nox2^fl/fl^CD4Cre^+^ compared with Nox2^fl/fl^ heart, vessels, and kidneys during Ang II infusion. This change in Treg/Th17 balance is accompanied by a higher level of IL-10 and a lower level of IL-17 in the hearts of Nox2^fl/fl^CD4Cre^+^ compared with Nox2^fl/fl^ mice after Ang II infusion, indicating a switch from a pro- to an antiinflammatory phenotype when Nox2 is deleted in Tregs. Our in vitro coculture studies suggest that the reduction in IL-17 levels is not related to a deficiency of Nox2 in Teffs but reflects the effects of Nox2-deficient Tregs (Figure 5H), consistent with the idea that there is a regulatory interplay between Tregs and Th17 cells (19).

In addition to an increase in CD4^+^CD25^+^ Tregs (conventional Tregs) in heart and aorta of Nox2^fl/fl^CD4Cre^+^ mice, we also observe an increased percentage of CD8^+^ Tregs in these tissues. This finding is most likely related to the CD4Cre-mediated targeting of Nox2 during the double-positive phase of maturation of these cells in the thymus. Indeed, double-positive CD4^+^CD8^+^ cells in Nox2^fl/fl^CD4Cre^+^ mice are found to be Nox2-deficient. These results suggest that Nox2 deficiency may have similar effects in conventional Tregs and CD8^+^ Tregs and that the latter cell type may also contribute to the effects observed in the current study. Nevertheless, the experiments with adoptive transfer of CD4^+^CD25^+^ Tregs clearly support the contention that Nox2 deficiency in conventional Tregs increases their suppressive ability and confers protection against Ang II–induced hypertension and cardiac fibrosis.

In the present study, we also find that the hearts of Nox2^fl/fl^CD4Cre^+^ mice express modestly higher levels of c-Met, CCR4, and CXCR3 mRNA than WT littermate hearts. Interestingly, CXCR3-positivity is reported to identify CD4^+^FoxP3^+^ Tregs with enhanced homing and suppressive capacity (45), while a recent study showed that a c-Met^+^CCR4^+^CXCR3^+^ phenotype is a specialized homing “signature” to instruct T cell cardiotropism (17). We speculate that these changes may contribute, at least in part, to the higher number of Tregs found in the hearts, aortae, and kidneys of Nox2^fl/fl^CD4Cre^+^ compared with control mice as well as the higher proportion of Nox2-deficient compared with WT Tregs found in the hearts of WT mice after adoptive transfer.

Previous studies showed that Nox2 expressed in cardiomyocytes, endothelial cells, and neurons contributes to the pathophysiology of Ang II–induced hypertension and cardiac remodeling (4–6, 46, 47). The involvement of Nox2-expressing T cells in Ang II–induced hypertension was suggested in a study in which the adoptive transfer of T cells into RAG-deficient mice (which lack T and B cells) restored hypertensive responses to Ang II, with full manifestation of this requiring T cells with a functional Nox2 (13). However, the role of different T cell subsets was not explored. Recently, it was reported that during Ang II–stimulated hypertension there is an accumulation of oxidatively modified isoketal-protein adducts in dendritic cells, which activate these cells and promote CD8^+^ T cell activation/proliferation and hypertension (48). Nox2 in dendritic cells may contribute to the formation of these oxidation products, and it was previously shown that dendritic cell Nox2 is involved in antigen presentation via MHC class II as a consequence of regulating phagosomal pH (49). In the current study, we identify, for the first time to our knowledge, a pivotal role of Nox2 in Tregs in regulating Ang II–induced hypertension and cardiac remodeling. Remarkably, Nox2 deletion solely in Tregs in adoptive transfer studies is sufficient to induce substantial inhibition of Ang II–induced hypertension and particularly interstitial cardiac fibrosis, pointing to the importance of Treg Nox2 in these processes. We observed increased numbers of Nox2-deficient Tregs in the heart, aorta, and kidney during Ang II infusion. It is likely that both vascular and renal Treg infiltration may contribute to the changes in Ang II–induced hypertension (although we do not find significant changes in renal function in Nox2^fl/fl^CD4Cre^+^ mice). The changes in Ang II–induced cardiac fibrosis and remodeling are likely to be due in large part to the direct effects of Tregs in the heart rather than secondary to the change in blood pressure, since cardiac fibrosis and remodeling are to a significant extent independent of hypertension in this model (Supplemental Figure 1).

In conclusion, this study uncovers a crucial role of Nox2 in CD4^+^CD25^+^FoxP3^+^ Tregs in regulating Ang II–induced hypertension and cardiac remodeling. The current results suggest that targeting Nox2 in Tregs might be a useful strategy in cardiovascular diseases. Given that Treg-based cell therapy is already in early clinical trials in transplant rejection (50), and that several approaches to Nox2 inhibition are feasible, studies to investigate the impact of Treg Nox2 in such pathological settings are also warranted.

## Methods

### Mice and in vivo studies.

All studies were performed in 6- to 8-week-old male mice on a C57BL/6J background. Globally Nox2-deficient mice (Nox2^–/y^) were originally acquired from The Jackson Laboratory. Nox2^fl/fl^CD4Cre^+^ mice were generated by crossing of CD4-Cre males (provided by R. Noelle, King’s College London) with Nox2^fl/fl^ females (6). Gene-modified mice were compared with matched WT littermates.

Ang II (1.1 mg/kg/d) was infused via subcutaneous osmotic minipumps (model 1002, Alzet) implanted under 2% isoflurane anesthesia. Blood pressure was determined by the tail cuff method (MK-2000ST, Muromachi Kikai). Echocardiography was performed in mice under 1.5% isoflurane anesthesia using a VisualSonics Vevo 2100 imaging system (FUJIFILM) (5). Renal function was assessed in response to an acute i.p. saline challenge in a metabolic cage (6). Metabolites were analyzed on an Advia 2400 Chemistry System (Siemens AG).

### Flow cytometry.

Hearts were perfused with 0.9% NaCl through the left ventricle before harvesting. Single-cell suspensions were prepared by digestion of heart, kidney, or aorta in a mixture of collagenase (1 mg/ml; catalog C5238, Sigma-Aldrich), DNase (160 IU/ml), and hyaluronidase (500 IU/ml) at 37°C for 30 minutes. Samples were triturated and sequentially filtered through a 40-μm nylon mesh. Red blood cells were lysed in 2% NH_4_Cl buffer. Nonspecific interactions were blocked with anti–mouse CD16/CD32 Ab (10 μg/ml, 1:50; catalog 14-0161-82, eBioscience) before staining. Anti-CD8–APC-Cy7, anti-CD45–FITC, anti-TCRβ–PE-Cy7, anti–MHC II–PerCP, anti-RORγT–BV421, anti-Nox2, anti-CD25–PE, anti–phospho-STAT5 Abs and anti-CD4 PercP were purchased from BD Pharmingen (catalog 561967, 553080, 560729, 562363, 562894, 611414, 553075, 562077, and 553052 respectively). Anti-CD4–eFluor450 and anti-FoxP3–APC Abs were acquired from eBioscience (catalog 48-0041-80 and 17-5773-82, respectively). Anti–rat Alexa Fluor 633 and anti–rabbit Alexa Fluor 488 were acquired from Invitrogen (catalog A21094 and A3273, respectively). The FoxP3 Transcription Factor Staining Buffer Set kit was from eBioscience (catalog 00-5523-00).

In order to determine the protein levels of CTLA-4, CD39, CD73, and GITR, Tregs were purified from lymph nodes and spleens using a Dynabeads FlowComp Mouse CD4^+^CD25^+^ Treg kit (Invitrogen). The mean fluorescence intensity corresponding to fluorochromes associated with CTLA-4, CD39, CD73, and GITR was evaluated in CD25^+^FoxP3^+^ cells. For this assay, anti-FoxP3–PE/Cy7 from Thermo Fisher Scientific (catalog 25-5773-82) and anti-CD25–PerCP-Cy5.5, anti–CTLA-4–APC, anti-GITR–FITC, anti-CD39–PE, and anti-CD73–Brilliant Violet 421 from Biolegend (catalog 102029, 106309, 126308, 143803, and 127217, respectively) were used.

Levels of phospho-STAT5 were evaluated in CD4^+^ T cells purified (>90%) from lymph nodes and spleens (Dynabeads Untouched Mouse CD4 Cells kit, catalog 11415D, Invitrogen). After stimulation with IL-2 (100 IU/ml), cells were fixed in 4% paraformaldehyde, stained with anti-CD4–eFluor450 and anti-CD25 PE-Cy7 Ab, incubated 30 minutes in permeabilization buffer (0.5% BSA, 0.5% saponin in PBS), and stained with FoxP3-APC and anti–phosho-STAT5–PE.

Samples were acquired in an LSRFortessa flow cytometer (BD Biosciences) and analyzed using FlowJo software 9.7.5.

### Adoptive transfer of purified Tregs.

Tregs were purified (>90%) from lymph nodes and spleens using a Dynabeads FlowComp Mouse CD4^+^CD25^+^ Treg kit. Immediately after purification, 1 × 10^6^ cells were injected in the tail vein, and the minipump containing Ang II was implanted afterward.

### Superoxide production.

Superoxide production was determined by flow cytometry using 10 μM dihydroethidium (6) in CD4^+^ T cells purified from Nox2^fl/fl^CD4Cre^+^ and Nox2^fl/fl^ mice and stimulated with anti-CD3 (2 μg/ml) plus anti-CD28 (4 μg/ml). Some cells were incubated with diphenyleneiodonium (1 μM) before TCR stimuli (negative controls).

### In vitro suppression assay.

Teffs (CD4^+^CD25^–^) and Tregs (CD4^+^CD25^+^) were purified from spleen and lymph nodes. APCs were obtained by incubation of total splenocytes with anti-CD4 and anti-CD8 Abs, followed by negative selection using Dynabeads from the Dynabeads Untouched Mouse CD4 Cells kit. Teffs were stained with CFSE (51) and incubated with Tregs under stimulation by anti-CD3ε (4 μg/ml) and APCs (1:2).

### Quantitative PCR.

RNA was extracted using RNeasy Mini Kits (Qiagen). After cDNA generation, SYBR Green real-time PCR was performed using the ΔΔCt method and β-actin for normalization. Primers were as follows: Nox2: forward ACTCCTTGGGTCAGCACTGG, reverse GTTCCTGTCCAGTTGTCTTCG; CCR4: forward ATCCTGAAGGACTTCAAGCTCCA, reverse AGGTCTGTGCAAGATCGTTTCATGG; c-Met: forward TCCTGCACTGTGAGCATTTC, reverse ACGATTGGGTTTCAGCAGAC; CXCR3: forward GTGGCTGCTGTGCTACTGAG, reverse AAGGCCCCTGCATAGAAGTT; β-actin: forward CTGTCGAGTCGCGTCCACCC, reverse ATGCCGGAGCCGTTGTCGAC; CD25: forward TGGTCTATATGCGTTGCTTGCTTAGG, reverse TTCTCGATTTGTCATGGGAGT; GITR: forward ATGAGGCCTGGTCTTCCTCT, reverse TTGTGCTAAACGTGGTGCTC; CTLA-4: forward TGGACCCTGAGCATCTCTCT, reverse CAGGTGTCTGCCTAGCCTTC; CD73: forward GCCTATGCCTTTGGCAAATA, reverse AGGTTTCCCATGTTGCATTC; CD39: forward CAAGGGCTGCGAGATAAGAC, reverse GCACCAGGGAACTTGGTAGA.

*NF-*κ*B activity*. Jurkat cells (clone EC6-1, ATCC TIB-152) were stimulated with Dynabeads Human T activator CD3/CD28 (1:1) for 12 hours. The cells were then transfected with NF-κB firefly luciferase (pGL4.32[luc2P/NF-κB-RE/Hygro] vector, Promega) and thymidine kinase Renilla luciferase constructs (phRL-TK, Promega) using electroporation in T cell nucleofector media (Lonza). Plasmids expressing minimal promoter firefly and thymidine kinase Renilla were used as controls. Luciferase activities in cell lysates were determined using the Dual-Glo luciferase system (Promega) in a plate luminometer (Mithras LB 940, Berthold).

### Immunofluorescence and ImageStream.

Levels of nuclear p65 and FoxP3 were evaluated in CD4^+^CD25^+^ T cells after stimulation with anti-CD3 (2 μg/ml) plus anti-CD28 (4 μg/ml). Cells were fixed with paraformaldehyde 4% and stained with anti-p65 Ab (catalog sc-372, Santa Cruz Biotechnology) and anti-mouse FoxP3 Ab (catalog 14-5773-82, eBioscience) and then with Alexa Fluor 488 anti-rabbit Ab and Alexa Fluor 633 anti-rat Ab. Nuclei were stained with DAPI (Sigma-Aldrich). Cell images were acquired by confocal microscopy (Leica TCS SP5).

Levels of nuclear FoxP3 and p65 and colocalization of FoxP3/p65 in CD4^+^ T cells purified from Nox2^–/y^ and WT mice were also determined using imaging flow cytometry. CD4^+^ T cells were stimulated with anti-CD3 (4 μg/ml) plus anti-CD28 (4 μg/ml) for 60 minutes and stained with anti-CD4–PE/Cy7 (catalog 25-0041-82, eBioscience) for 30 minutes. Cells were then fixed, permeabilized, and stained with anti-FoxP3–APC, rabbit anti-p65, DAPI, and goat Alexa Fluor 488 anti-rabbit Ab. Cell images were acquired in ImageStreamX MKII (Merck Millipore) and analyzed using IDEAS 6.2 software (NIH). Nuclear translocation and colocalization wizards available in the software guided the analyses. Parameters were evaluated in CD4^+^FoxP3^+^ cells (500–5,000 cells per group). The coefficient of similarity (Cs) was defined as “Similarity” or “Bright field similarity” in channels corresponding to FoxP3, DAPI, and p65.

### Cytokine production and immunoblotting.

Levels of TNF-α and IFN-γ were determined in supernatants of cocultures of Teffs and Tregs using a Cytometric Bead Array kit (BD Pharmingen). Levels of IL-17 and IL-10 were determined by ELISA using kits from R&D Systems and eBioscience, respectively.

Nuclear protein from purified Tregs was immunoblotted for FoxP3 (catalog 145773-82, eBioscience) and histone H3 (catalog 4499S, Cell Signaling Technology) using standard methods. Anti-rabbit 680VW and anti-rat 800VW Licor Abs (catalog 925-68071 and 925-32219, respectively) were used as secondary Abs. Signals were analyzed using the Odyssey CLx infrared Imaging System.

### Histology.

Leukocyte infiltration, cardiomyocyte hypertrophy, and fibrosis were analyzed in paraffin-embedded sections stained with H&E, wheat germ agglutinin, and Picrosirius red, respectively. Cryosections were used for CD3 immunostaining. Images were acquired on a DM200 LED bright-field or confocal TCS SP5 (Leica) microscope. ImageJ software (NIH) was used for analyses.

### Statistics.

Analyses were performed using GraphPad Prism software 5.0 (GraphPad Software Inc.). Data are reported as mean ± SEM. Comparisons were undertaken using unpaired 2-tailed *t* test or 1-way or 2-way ANOVA followed by Tukey’s post-test, as appropriate. *P* less than 0.05 was considered significant.

### Study approval.

All procedures in animals were undertaken in accordance with the Guidance on the Operation of the Animals (Scientific Procedures) Act, 1986 (UK Home Office), and with institutional ethics approval from King’s College, London, United Kingdom.

## Author contributions

AMS and GL conceived and supervised the study. AE, HMD, and SCT contributed to experimental design. AE, HMD, SCT, AI, LAS, RE, QP, CO, GS, and PDB performed experiments and interpreted data. RIL provided critical intellectual input. AE, SCT, HMD, GL, and AMS wrote the manuscript.

## Supplementary Material

Supplemental data

## Version 1 04/24/2018

In-Press Preview

## Version 2 06/11/2018

Electronic publication

## Version 3 07/02/2018

Print issue publication
