# Supplementary material for: Nox2 in regulatory T cells promotes angiotensin II–induced cardiovascular remodeling
Source: J Clin Invest. 2018 Jun 11;128(7):3088–101. doi: 10.1172/JCI97490 (PMC6025997; doi:10.1172/JCI97490)
Supplement: Supplemental data [file jci-128-97490-s278.pdf]

Nox2 in regulatory T cells promotes angiotensin II-induced cardiovascular remodeling

Amber Emmerson[1†], Silvia Cellone Trevelin[1†], Heloise Mongue-Din[1†], Pablo D. Becker[2], Carla Ortiz[2], Lesley A. Smyth[2], Qi Peng[2], Raul Elgueta[2], Greta Sawyer[1], Aleksandar Ivetic[1], Robert I. Lechler[2], Giovanna Lombardi[2*], Ajay M Shah[1*]

[1]King's College London British Heart Foundation Centre, School of Cardiovascular Medicine and Sciences, London, United Kingdom; [2]King's College London, Division of Transplantation Immunology & Mucosal Biology, London, United Kingdom. [†]These authors contributed equally to this study. [*]Professor Giovanna Lombardi and Professor Ajay M Shah are co-senior authors.

***Corresponding authors:** Professor Ajay M. Shah, Department of Cardiology, James Black Centre, 125 Coldharbour Lane, London SE5 9NU, UK. Tel: 0044-207848-5189; Fax: 0044-207848-5193. Email: ajay.shah@kcl.ac.uk; **and** Professor Giovanna Lombardi, Immunoregulation Laboratory, MRC Centre for Transplantation, King's College London, 5th Floor Tower Wing, Guys' Hospital, London SE1 9RT, UK. Email: giovanna.lombardi@kcl.ac.uk.

The authors have declared that no conflicts of interest exist.

Running title: Treg Nox2 and cardiovascular remodeling

Total character count: 56,145

This manuscript requires a Creative Commons CC-BY license.

Supplemental Figures

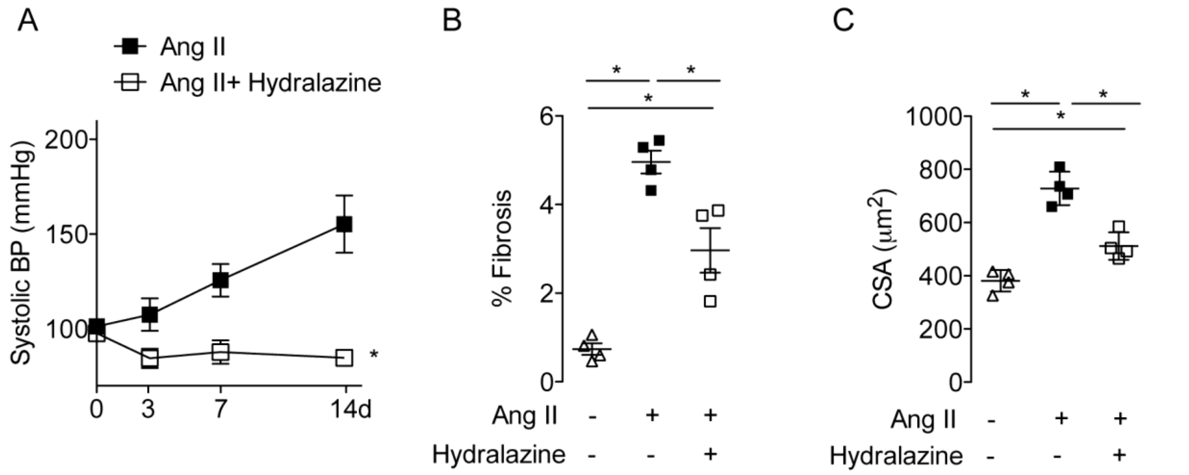

Supplemental Figure 1

Supplemental Figure 1. Effect of hydralazine on the response to chronic Angiotensin II (Ang II) infusion. WT mice receiving chronic Ang II infusion (1.1mg/kg/day by osmotic minipump) were treated with hydralazine (300 mg/L in drinking water) to normalise BP. (A) Systolic BP (BP) over 14 days of Ang II infusion. (B) % interstitial cardiac fibrosis in myocardial sections (Picrosirius Red staining). (C) Cardiomyocyte cross-sectional area (CSA) in transverse myocardial sections. Data are represented as mean±SEM *$P<0.05$* by 2-way ANOVA (A) or 1-way ANOVA followed by Tukey's post-test (B-C); n=4 per group.

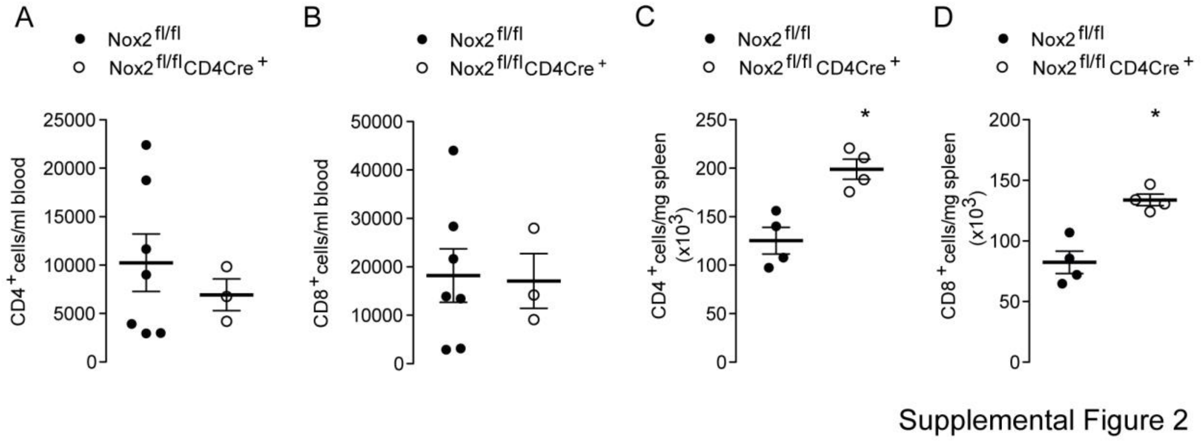

Supplemental Figure 2. Circulating and splenic CD4^{+} and CD8^{+} T cells in Nox2$^{fl/fl}$CD4Cre^{+} mice compared to Nox2$^{fl/fl}$ controls. (A, B) Flow cytometry analyses of circulating CD4^{+} and CD8^{+} T cells. (C, D) Flow cytometry analyses of CD4^{+} and CD8^{+} T cells in spleen. Data are represented as mean±SEM *$P<0.05$ by unpaired t-test; n=4-7 per group.

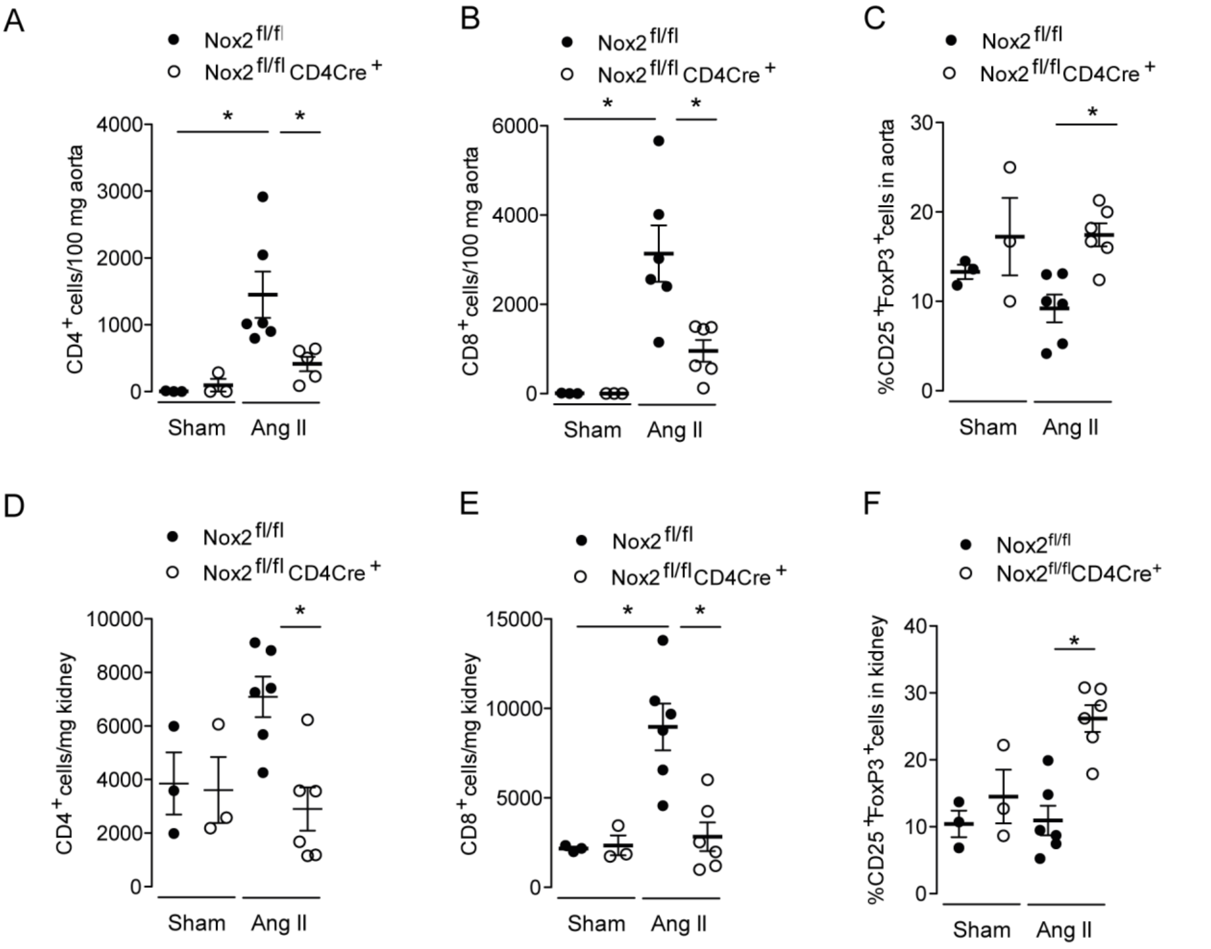

Supplemental Figure 3

Supplemental Figure 3. Numbers of Tregs and $CD4^+$ and $CD8^+$ Teffs in aorta and kidneys of $Nox2^{fl/fl}CD4Cre^+$ mice treated with AngII. $Nox2^{fl/fl}CD4Cre^+$ and $Nox2^{fl/fl}$ controls were treated with AngII (1.1mg/kg/day) or saline (Sham) infusion. Number of $CD45^+TCR\beta^+CD4^+$ (A, D) and $CD45^+TCR\beta^+CD8^+$ T cells (B, E) in aorta and kidney digests by flow cytometry after 7 days of Ang II treatment. (C, F) Relative numbers of Tregs in aorta and kidney digests after 7 days of Ang II treatment. Data are represented as mean±SEM. *$P<0.05$ by 1-way ANOVA followed by Tukey's post-test; n=3-6 per group.

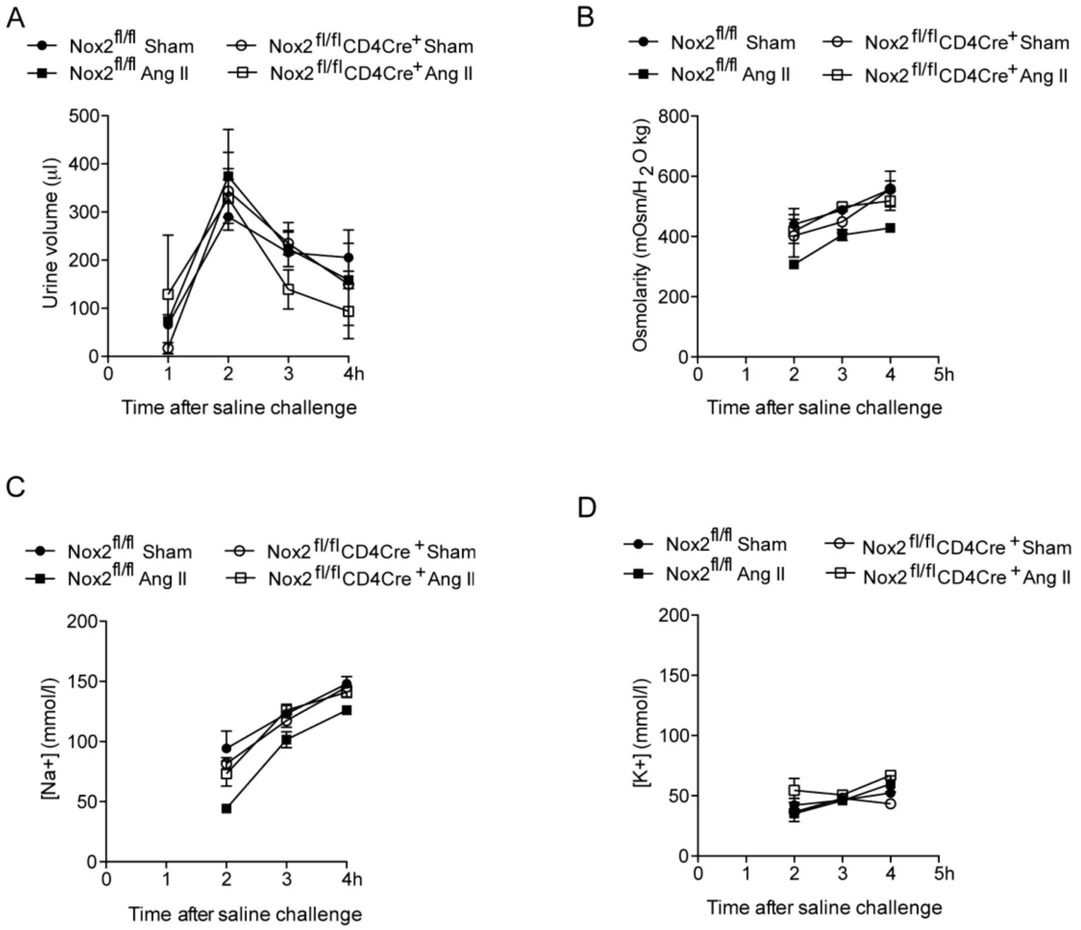

Supplemental Figure 4

Supplemental Figure 4. Renal function is not altered in Nox2$^{fl/fl}$CD4Cre^{+} mice after AngII infusion. Mice were treated with AngII (1.1mg/kg/day) or saline (Sham) infusion. Renal function was assessed in response to an acute saline challenge (40 ml/kg 0.9% w/vol. saline, i.p.). Animals were placed in individual metabolic chambers for 4 hours and urine was collected hourly. Data are represented as mean±SEM. **P<0.05* by 2-way ANOVA; n=3 per group.

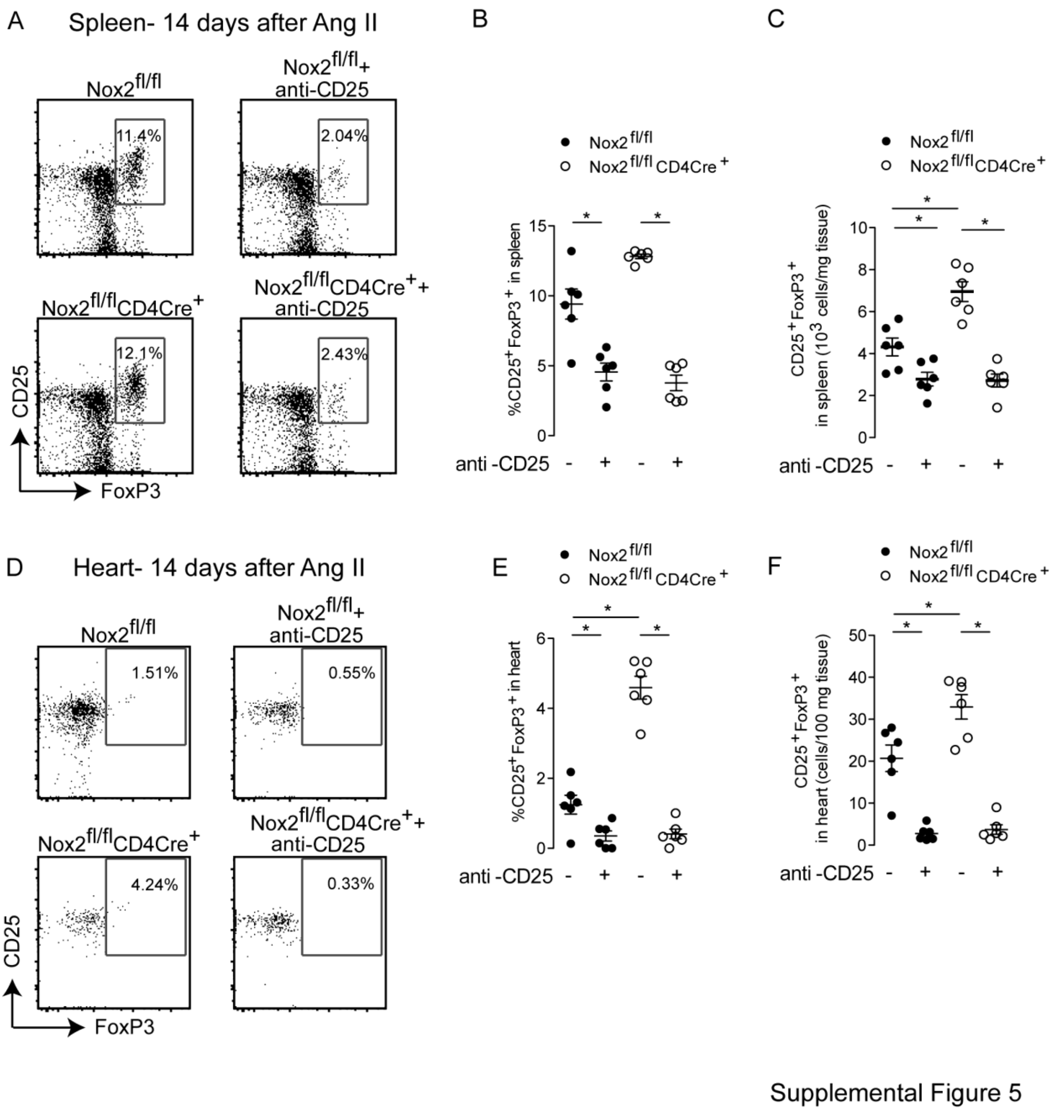

Supplemental Figure 5. Anti-CD25 Ab treatment reduces numbers of Tregs in spleen and hearts of Nox2$^{fl/fl}$CD4Cre^{+} and Nox2$^{fl/fl}$ littermate controls. Nox2$^{fl/fl}$CD4Cre^{+} and Nox2$^{fl/fl}$ littermate controls mice were treated with Ang II infusion (1.1mg/kg/day). Some mice were treated with anti-CD25 antibody (Clone PC61, 500µg/mouse, i.p.) one day before minipump implantation. Relative and absolute numbers of CD45^{+}TCR^{+}CD4^{+}CD25^{+}FoxP3^{+} cells (Tregs) in spleen (A-C) and heart (D-F) after 14 days of Ang II treatment. Data are represented as mean±SEM. *$P<0.05$ by 1-way ANOVA followed by Tukey's post-test; n=6 per group.

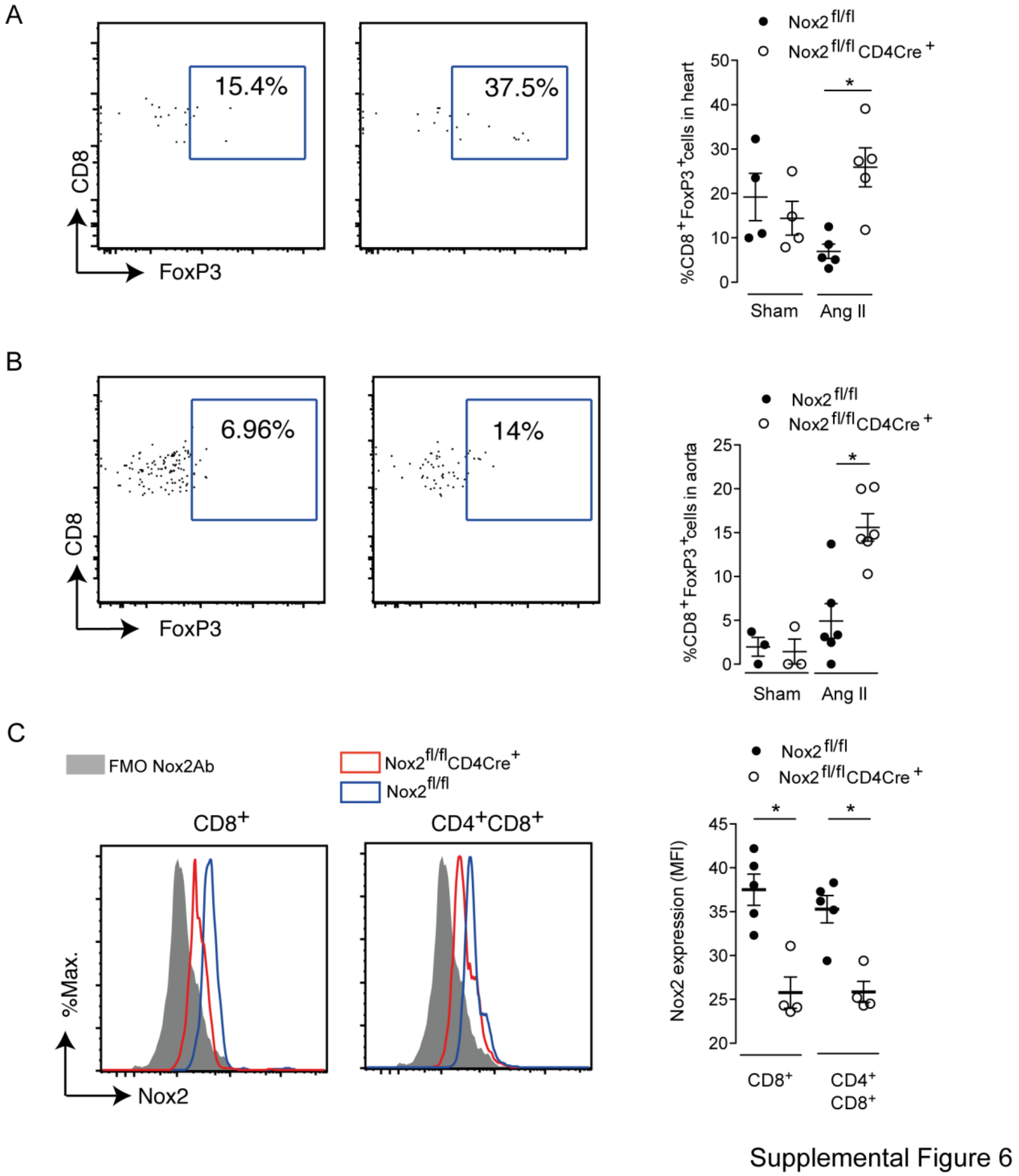

Supplemental Figure 6

Supplemental Figure 6. Nox2$^{fl/fl}$CD4Cre^{+} mice have an increased proportion of CD8^{+}FoxP3^{+} T cells in heart and aorta after AngII treatment. Nox2$^{fl/fl}$CD4Cre^{+} and Nox2$^{fl/fl}$ littermate controls were treated with AngII (1.1mg/kg/day) or saline (Sham) infusion. Relative numbers of CD8^{+} Tregs in heart (A) and aorta (B) digests after 7 days of Ang II treatment. (C) Nox2 expression in CD8^{+} T cells or CD4^{+}CD8^{+} cells from thymus. Data are represented as mean±SEM. The histogram representing FMO Nox2Ab control is the same in both panels. **P<0.05* by 1-way ANOVA followed by Tukey's post-test; n=3-6 per group.

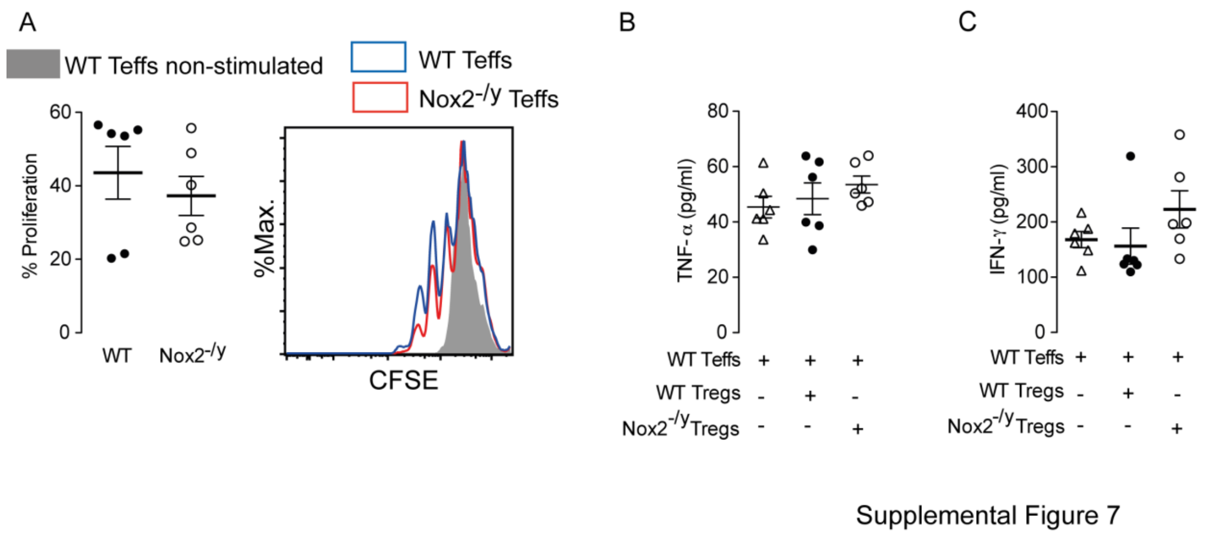

Supplemental Figure 7. Nox2 deficiency does not change the production of TNF-α and IFN-Y by effector T cells. $CD4^+CD25^-$ cells (Teff) were co-cultured with Tregs ($CD4^+CD25^+$ cells) purified from spleen and lymph nodes of Nox2-deficient mice ($Nox2^{-/y}$) and WT controls. Cells were stimulated with antigen presenting cells and anti-CD3ε antibody (4μg/ml) for 3 days. (A) % proliferation of Teffs; a representative histogram of WT and $Nox2^{-/y}$ Teffs proliferation is shown to the right. (B) Levels of TNF-α and IFN-γ were determined by cytometric bead array in culture supernatants. Data are represented as mean±SEM. Groups were compared by unpaired t-test (A) or 1-way ANOVA followed by Tukey's post-test (B, C); n=6 per group.

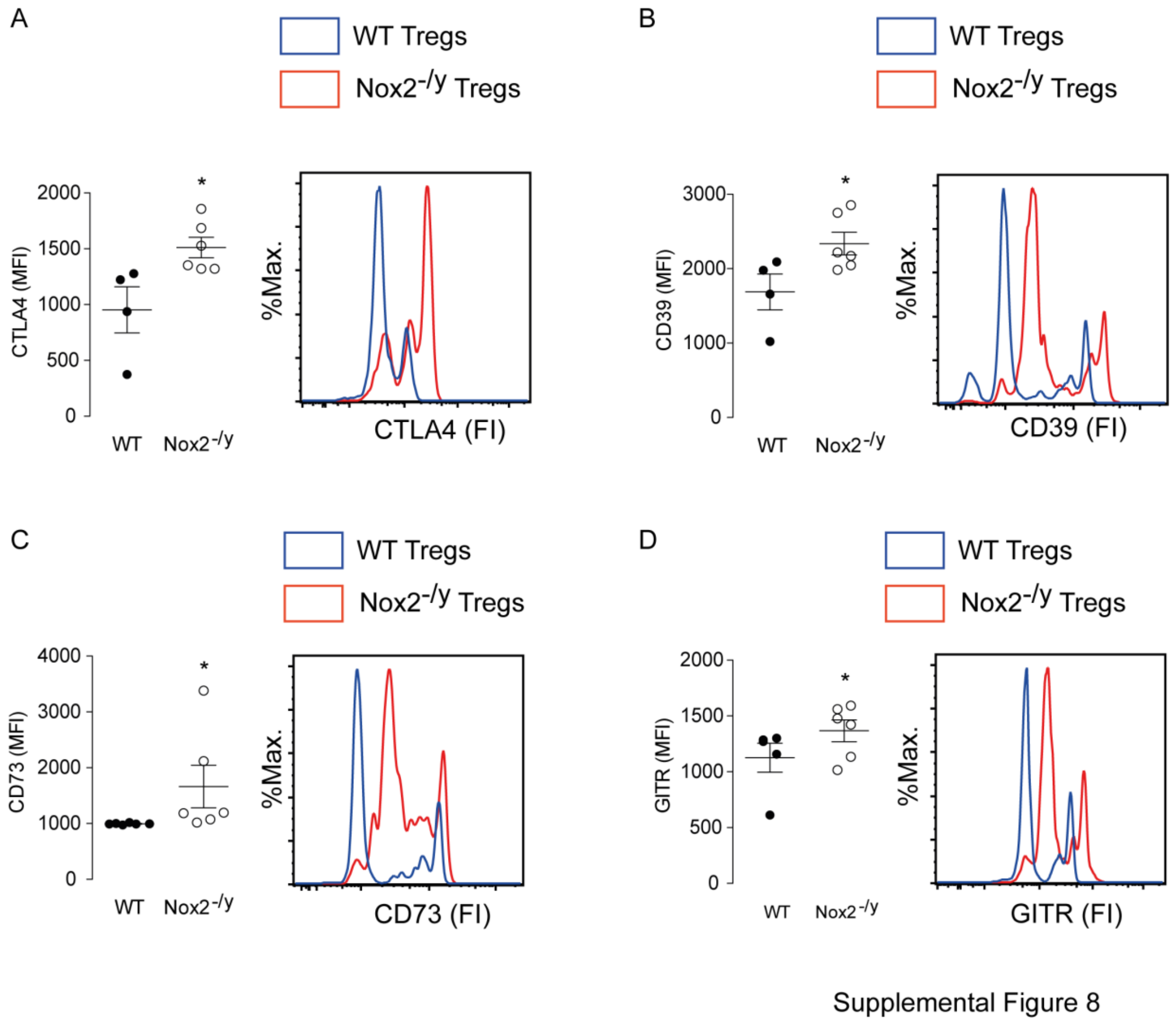

Supplemental Figure 8. Nox2 deficiency in Tregs enhances protein levels of suppressive molecules. The protein levels of CTLA4, CD39, CD73 and GITR were quantified in purified Tregs ($CD4^+CD25^+FoxP3^+$ cells) by flow cytometry. MFI, mean fluorescence intensity; FI, fluorescence intensity. Data are represented as mean±SEM. **P<0.05* by unpaired t-test; n=4-6 per group.

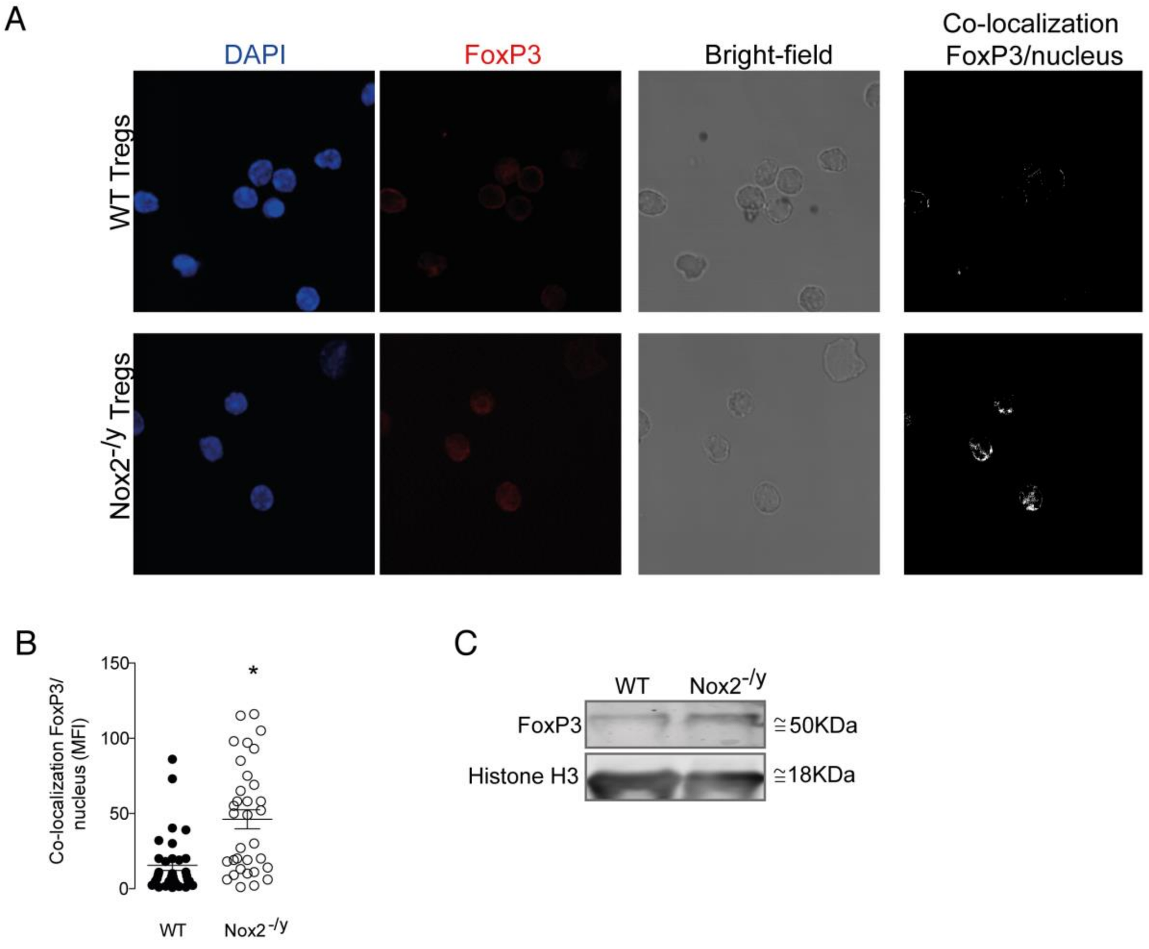

Supplemental Figure 9

Supplemental Figure 9. Nox2 deficient Tregs have increased nuclear levels of FoxP3. (A, B) Localization of FoxP3 in the nucleus, evaluated by confocal microscopy. Co-localized points were determined using the plug-in “co-localization highlighting” in Image J software. MFI (mean fluorescence intensity). Representative images are shown in A and mean data in B. Data are represented as mean±SEM. *$P<0.05$ by unpaired t-test; n=33 cells per group. (C) Levels of FoxP3 in the nuclear fraction of Tregs by immunoblot. Histone H3 was used as a loading control.
